# Associations between triglyceride glucose-body mass index and cardiovascular, renal, hepatic and bone biomarkers in patients with type 2 diabetes: a single-center, cross-sectional study

**DOI:** 10.3389/fendo.2025.1566818

**Published:** 2025-09-12

**Authors:** Nan Xu, Kunyi Wu, Ting La, Ruo Zhang, Bo Cao

**Affiliations:** ^1^ Department of Clinical Laboratory, The Second Affiliated Hospital of Xi’an Jiaotong University, Xi’an, Shaanxi, China; ^2^ Core Research Laboratory, The Second Affiliated Hospital of Xi’an Jiaotong University, Xi’an, Shaanxi, China; ^3^ National-Local Joint Engineering Research Center of Biodiagnosis and Biotherapy, The Second Affiliated Hospital of Xi’an Jiaotong University, Xi’an, Shaanxi, China; ^4^ Department of Endocrinology, The Second Affiliated Hospital of Xi’an Jiaotong University, Xi’an, Shaanxi, China

**Keywords:** type 2 diabetes, TyG-BMI, cardiovascular biomarker, renal biomarker, hepatic biomarker, bone biomarker

## Abstract

**Background:**

Type 2 diabetes (T2D) is a prevalent chronic metabolic disorder with significant global health implications, characterized by elevated blood glucose levels and a cluster of cardiometabolic risk factors. The triglyceride glucose-body mass index (TyG-BMI) has emerged as a novel index for assessing insulin resistance (IR) and cardiometabolic risk in T2D patients.

**Objective:**

This study aims to investigate the associations between TyG-BMI and the characteristics of cardiovascular, renal, hepatic and bone biomarkers in T2D patients, particularly in the context of Cardiovascular-Kidney-Metabolic (CKM) syndrome.

**Methods:**

We conducted a single-center, cross-sectional study involving 2,981 T2D patients. We assessed TyG-BMI and its correlations with the incidence of cardiovascular events, as well as bone, renal, and hepatic biomarkers. The study included 29 clinical characteristics of T2D patients to understand the progressive nature of T2D complications and the potential of TyG-BMI as a risk assessment tool.

**Results:**

Preliminary findings suggest a strong association between elevated TyG-BMI and increased trends of cardiovascular, renal, and hepatic, and bone biomarkers in T2D patients. The TyG-BMI showed significantly different trends among quartile subgroups for all identified key assessing indicators, indicating its potential as a convenient and effective tool for risk assessment.

**Conclusion:**

The TyG-BMI index is potentially associated with cardiovascular, renal, hepatic, and bone biomarkers in T2D patients. These findings could contribute to the development of strategies for the prevention and translational therapies of related complications in T2D patients, ultimately improving patient outcomes and reducing the burden on healthcare systems.

## 1 Introduction

Type 2 diabetes (T2D) is a chronic metabolic disorder with a rapidly increasing prevalence in recent years and has posed a significant global health challenge for public health worldwide (1–3). T2D is not only characterized by elevated blood glucose levels but also by a cluster of cardiometabolic risk factors, including dyslipidemia, hypertension, and obesity, which contribute to the development of microvascular and macrovascular complications (4–6). These complications, which include heart disease, stroke, kidney disease, nerve damage, and eye problems, significantly impact the quality of life of patients and impose a substantial burden on healthcare systems (7–10).

The triglyceride glucose-body mass index (TyG-BMI), a novel index that combines fasting triglyceride levels, fasting glucose, and body mass index, has emerged as a potentially powerful tool for assessing insulin resistance (IR) and cardiometabolic risk in T2D patients (11–13). This index provides a simple and feasible alternative to more complex and resource-intensive methods, offering a comprehensive assessment of cardiometabolic risk (14–16). Previous studies have demonstrated a close relationship between the TyG-BMI index and IR, as well as its association with cardiovascular diseases (CVD) and related outcomes in acute coronary syndrome, heart failure, and ischemic stroke (17, 18).

Specifically, the association between TyG-BMI and the risks of cardiovascular, renal, hepatic and bone biomarkers in patients with T2D is of particular interest. Emerging evidences suggest that TyG-BMI may play a significant role in predicting prognosis in patients with T2D and coronary heart disease (CHD) (19, 20). Furthermore, the TyG-BMI index has been shown to be positively associated with the incidence of cardiovascular events in populations with Cardiovascular-Kidney-Metabolic (CKM) syndrome stages 0–3, highlighting its potential as a convenient and effective tool for risk assessment in these populations (21).

Given the importance of these biomarkers in the development of T2D, it is crucial to investigate the associations between TyG-BMI and cardiovascular, renal, hepatic and bone complication characteristics in T2D population. This will aid in the study of progressive T2D and the implementation of early and comprehensive intervention strategies, ultimately aiming to reduce the burden of a series of severe complications for T2D patients.

Based on the above assumptions, in this study we conducted a single-center, cross-sectional study involving 2,981 T2D patients to investigate the associations between TyG-BMI and the characteristics of cardiovascular, renal, hepatic and bone biomarkers. By examining these associations, we hope to gain insights into the role of triglyceride metabolism in the development of T2D complications and to identify potential targets for therapeutic intervention. Our findings will contribute to develop strategies for the prevention and management of complications in T2D patients, ultimately improving patient outcomes and reducing the burden on healthcare systems.

## 2 Materials and methods

### 2.1 Study design

This single-center, cross-sectional study analyzed baseline data from 2,981 T2D patients (1,883 male and 1,098 female) admitted to the Second Affiliated Hospital of Xi’an Jiaotong University from July 2021 to July 2024. No longitudinal follow-up was performed. This study conforms to the principles of the Declaration of Helsinki and adheres to the Good Clinical Practice guidelines. The study protocol, including any amendments, and associated documents were granted approval by the relevant institutional review boards, ethical committees, and regulatory entities (No. 2021106).

### 2.2 Inclusion and exclusion criteria

Inclusion criteria: All patients must satisfy the diagnostic criteria for T2D, which includes experiencing diabetic symptoms alongside one of the following: 1) fasting blood glucose ≥ 7.0 mmol/L (126 mg/dL); 2) random blood glucose or 2-hour post-challenge glucose ≥ 11.1 mmol/L (200 mg/dL); 3) HbA1c ≥ 6.5% (48 mmol/mol).

Exclusion criteria: 1) individuals with type 1 diabetes (T1D) or other specific forms of diabetes; 2) patients who have experienced a myocardial infarction, cerebral hemorrhage, severe hepatic or renal impairment, acute infections, or significant stress within the last three months; 3) pregnant or breastfeeding women; 4) patients diagnosed with cancers or mental health conditions that may hinder their ability to adhere to study protocols; 5) those who have taken relevant medications that impact cardiovascular (RAAS inhibitors, β-blockers), renal (SGLT2 inhibitors, NSAIDs), hepatic (Statins, hepatotoxic drugs [allopurinol > 100 mg/day]) and bone (Bisphosphonates, teriparatide, vitamin D > 2000 IU/day) metabolisms within the past three months at the time of testing.

### 2.3 Clinical data

**Figure 1:**
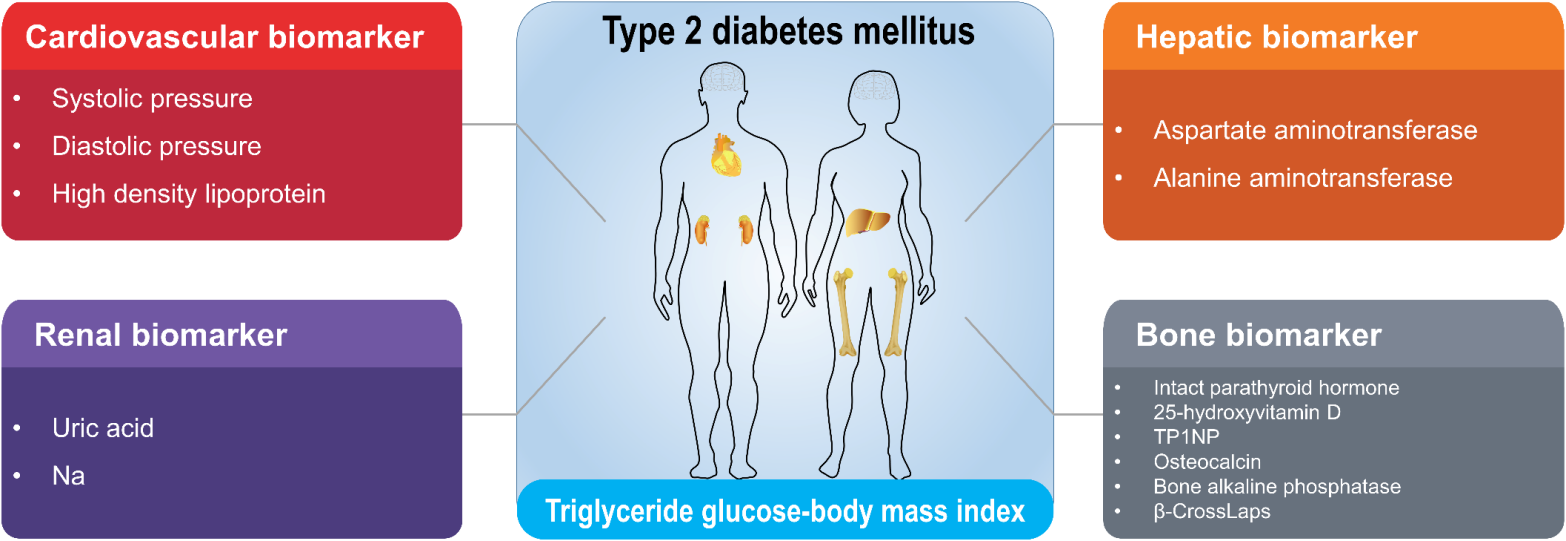
Classification of cardiovascular, renal, hepatic and bone biomarkers correlated with TyG-BMI in T2D patients. The clinical biomarkers with high correlations with TyG-BMI in T2D patients were identified and classified into cardiovascular, renal, hepatic and bone categories. The full names for abbreviations of clinical biomarkers are: TP1NP, Total procollagen type 1 N-terminal propeptide; Na, sodium.


The clinical data of T2D patients used in this study were estimated and deposited in the Department of Laboratory of the Second Affiliated Hospital of Xi’an Jiaotong University. Basic information including sex, age and course of disease was recorded after admission. Meanwhile, a total of 26 clinical characteristics including triglycerides (TG), fasting blood glucose, body mass index (BMI), HbA1c, systolic pressure, diastolic pressure, intact parathyroid hormone, 25-hydroxyvitamin D, total procollagen type 1 N-terminal propeptide (TP1NP), osteocalcin, bone alkaline phosphatase, β-CrossLaps, albumin, aspartate aminotransferase (AST), alanine aminotransferase (ALT), total cholesterol, high density lipoprotein (HDL), low density lipoprotein (LDL), creatinine, uric acid, eGFR-EPI (estimating glomerular filtration rate using the chronic kidney disease epidemiology collaboration [CKD-EPI] equation), urea, sodium (Na), potassium (K), CO_2_, and calcium (Ca) were detected. Based on their physiological roles as well as functions in the progression of T2D, we further assessed and classified the above biomarkers related to T2D complications into four categories: cardiovascular, renal, hepatic, and bone biomarkers (**Figure 1**; **Supplementary Table S1**). For cardiovascular complication, we identified three key biomarkers: systolic pressure, diastolic pressure, and high-density lipoprotein. Regarding renal complication, we identified two significant biomarkers: uric acid levels and sodium levels. For hepatic complication, two relevant biomarkers were AST and ALT. Lastly, we identified six biomarkers related to bone complication: intact parathyroid hormone, 25-hydroxyvitamin D, TP1NP, osteocalcin, bone alkaline phosphatase, and β-CrossLaps. These selected bone markers reflect key turnover pathways: TP1NP/osteocalcin (formation), β-CrossLaps (resorption), and 25(OH)D/IPTH (mineral metabolism). The characteristic of TyG-BMI was calculated as following:

$$\text{TyG}-\text{BMI}=\ln(\frac{\text{fasting blood glucose }(\text{mg}/\text{dL})\times \text{TG }(\text{mg}/\text{dL})\;}{2})\times \frac{\text{body weight }(\text{kg})}{\text{height }{\left(\text{m}\right)}^{2}\;}$$

### 2.4 Statistical analysis

The data in this study were recorded and processed using Microsoft Excel 2016. Data were analyzed using IBM SPSS Statistics 23. The normality and homogeneity of variance for continuous data were assessed. The distribution normality was evaluated using Kolmogorov-Smirnov test. Data that were normally distributed are presented as mean ± standard deviation, and group comparisons were conducted using one-way ANOVA (analysis of variance). For non-normally distributed data, results are presented as median (interquartile range), and nonparametric tests were utilized. Count data are expressed as numbers (percentages), with the chi-square test applied for comparisons between groups. Pearson correlation analysis was used to examine the relationships among clinical characteristics. *P* < 0.05 was considered statistically significant based on a two-tailed test. Data were plotted using GraphPad Prism 9 software.

## 3 Results

### 3.1 Clinical characteristics of T2D patients in this study

The baseline characteristics of the study population reflect a representative sample of patients with T2D (**Table 1**). In general, the study cohort consisted of 2,981 patients, with a mean age of 58 ± 12 years. The majority of patients were male (63.2%), compared to female with a percentage of 36.8%. The median duration of disease was 10 years (interquartile range [IQR]: 4, 15), with a range from 0 to 46 years. The mean TyG-BMI value for the entire cohort was 227 ± 42, with a median of 224 (IQR: 199, 250). The range of TyG-BMI values spanned from 13 to 560, indicating a wide variation among participants. The other key clinical characteristics were as following.

**Table 1:** Baseline clinical characteristics of T2D patients included in this study.

Characteristic (N = 2,981)	Mean ± SD	Median (IQR)	Range	95% CI
Sex				
Female	1,098 (36.8%)			35%, 39%
Male	1,883 (63.2%)			61%, 65%
Age (year)	58 ± 12	59 (51, 67)	14, 90	58, 58
Course of disease (year)	10 ± 7	10 (4, 15)	0, 46	10, 11
TyG-BMI	227 ± 42	224 (199, 250)	13, 560	226, 229
TG (mg/dL)	173 ± 187	125 (87, 194)	30, 4,237	167, 180
Fasting blood glucose (mg/dL)	166 ± 86	147 (122, 193)	50, 3,402	163, 169
BMI (kg/m^2^)	24.5 ± 3.4	24.3 (22.3, 26.4)	1.5, 45.2	24, 25
HbA1c (%)	8.38 ± 2.63	7.90 (6.80, 9.52)	4.70, 98.70	8.3, 8.5
Systolic pressure (mmHg)	133 ± 19	132 (120, 146)	58, 208	132, 134
Diastolic pressure (mmHg)	84 ± 11	84 (76, 91)	43, 146	84, 85
Intact parathyroid hormone (pg/mL)	46 ± 26	41 (32, 53)	5, 476	45, 47
25-hydroxyvitamin D (µg/L)	18 ± 8	16 (12, 22)	3, 70	17, 18
TP1NP (ng/mL)	41 ± 31	36 (28, 48)	6, 1,200	40, 42
Osteocalcin (ng/mL)	12.9 ± 7.0	11.7 (9.1, 15.1)	0.6, 138.2	13, 13
Bone alkaline phosphatase (ng/mL)	16 ± 8	14 (11, 19)	1, 95	16, 16
β-CrossLaps (ng/mL)	0.40 ± 0.24	0.35 (0.25, 0.50)	0.06, 2.72	0.39, 0.41
Albumin (g/L)	44.7 ± 4.0	45.1 (42.7, 47.2)	8.0, 72.2	45, 45
Aspartate aminotransferase (IU/L)	22 ± 14	19 (15, 24)	5, 315	21, 22
Alanine aminotransferase (IU/L)	24 ± 22	19 (14, 27)	1, 482	23, 25
Total cholesterol (mmol/L)	4.30 ± 1.20	4.22 (3.47, 4.98)	1.26, 23.75	4.3, 4.3
High density lipoprotein (mmol/L)	1.21 ± 0.30	1.17 (1.00, 1.38)	0.43, 2.83	1.2, 1.2
Low density lipoprotein (mmol/L)	2.48 ± 0.94	2.44 (1.77, 3.07)	0.13, 7.42	2.4, 2.5
Creatinine (µmol/L)	74 ± 43	68 (58, 80)	23, 1,024	72, 75
Uric acid (µmol/L)	320 ± 89	311 (259, 373)	5, 825	317, 324
eGFR-EPI (mL/min/1.73m^2^)	93 ± 23	96 (84, 105)	4, 455	92, 94
Urea (mmol/L)	5.85 ± 2.16	5.49 (4.59, 6.70)	0.81, 30.58	5.8, 5.9
Na (mmol/L)	138.95 ± 2.67	139.05 (137.40, 140.80)	122.90, 151.90	139, 139
K (mmol/L)	4.16 ± 0.34	4.10 (3.90, 4.30)	2.50, 6.10	4.1, 4.2
CO_2_ (mmol/L)	21.41 ± 2.74	21.20 (19.60, 23.00)	4.90, 30.70	21, 22
Ca (mmol/L)	2.29 ± 0.11	2.29 (2.23, 2.36)	1.57, 2.80	2.3, 2.3

The full names of abbreviations are: TG, Triglycerides; BMI, Body mass index; TP1NP – Total procollagen type 1 N-terminal propeptide; eGFR-EPI, Estimating glomerular filtration rate using the chronic kidney disease epidemiology collaboration (CKD-EPI) equation; Na, sodium; K, potassium; Ca, calcium.

In terms of cardiovascular indicators, the systolic pressure of the study population was found to have a mean of 133 mmHg with a standard deviation of 19 mmHg. The median value was 132 mmHg, and the IQR was from 120 to 146 mmHg. The range of systolic pressure values observed in the study was from 58 to 208 mmHg, with a 95% confidence interval (CI) of 132 to 134 mmHg. While for diastolic pressure, it had a mean of 84 mmHg with a standard deviation of 11 mmHg. The median value was 84 mmHg, and the IQR was from 76 to 91 mmHg. The range of diastolic pressure values was from 43 to 146 mmHg, with a 95% CI of 84 to 85 mmHg.

As for renal indicators, the mean uric acid level was 320 ± 89 µmol/L, with a median of 311 µmol/L. The range of uric acid levels spanned from 5 to 825µmol/L, indicating a wide variability in uric acid concentrations among the study population. Elevated uric acid levels can be indicative of reduced renal function or other underlying health conditions that may contribute to renal risk. The mean sodium (Na) level was 138.95 ± 2.67 mmol/L, with a median of 139.05 mmol/L. Sodium levels ranged from 122.90 to 151.90 mmol/L, suggesting that the majority of patients had sodium levels within a normal range.

For hepatic indicators, the mean aspartate aminotransferase (AST) level was 22 ± 14 IU/L, with a median of 19 IU/L. The range of AST levels extended from 5 to 315 IU/L, indicating a large variability in AST concentrations among the study population. The mean alanine aminotransferase (ALT) level was 24 ± 22 IU/L, with a median of 19 IU/L. ALT levels ranged from 1 to 482 IU/L, showing a similar variability to AST levels. ALT is primarily found in the liver and is a more specific marker of liver cell damage.

While for bone indicators, the mean level of intact parathyroid hormone (IPTH) was recorded at 46 ± 26 pg/mL, with a median value of 41 (IQR: 32, 53) and a range from 5 to 476 pg/mL. IPTH is a critical regulator of calcium and phosphate metabolism, and its levels are frequently elevated in conditions such as hyperparathyroidism and renal insufficiency. Similarly, the mean concentration of 25-hydroxyvitamin D (25[OH]D) was determined to be 18 ± 8 µg/L, with a median of 16 (IQR: 12, 22) and a range from 3 to 70 µg/L. Vitamin D is essential for maintaining bone health, with levels below 20 µg/L generally classified as deficient, potentially leading to osteoporosis and an increased risk of fractures. The mean level of the bone formation marker total procollagen type 1 N-terminal propeptide (TP1NP) was found to be 41 ± 31 ng/mL, with a median of 36 (IQR: 28, 48) and a range from 6 to 1200 ng/mL. TP1NP serves as an important biomarker for assessing the rate of bone synthesis. The mean concentration of osteocalcin, another key indicator of bone formation produced by osteoblasts, was measured at 12.9 ± 7.0 ng/mL, with a median of 11.7 (IQR: 9.1, 15.1) and a range from 0.6 to 138.2 ng/mL. Furthermore, the mean level of bone alkaline phosphatase was 16 ± 8 ng/mL, with a median of 14 (IQR: 11, 19) and a range from 1 to 95 ng/mL. This enzyme serves as a marker of bone turnover and is typically elevated in conditions characterized by increased bone formation or resorption. Finally, the mean concentration of β-CrossLaps was 0.40 ± 0.24 ng/mL, with a median of 0.35 (IQR: 0.25, 0.50) and a range from 0.06 to 2.72 ng/mL. β-CrossLaps is utilized as a marker of bone resorption, providing insights into the process of bone degradation.

Collectively, the above results provide a comprehensive overview of the demographic and clinical characteristics of the patient cohort, highlighting the diversity in age, sex, and metabolic profiles, which are crucial for understanding the study population and interpreting the subsequent analyses.

### 3.2 Associations among clinical characteristics in T2D patients

**Figure 2:**
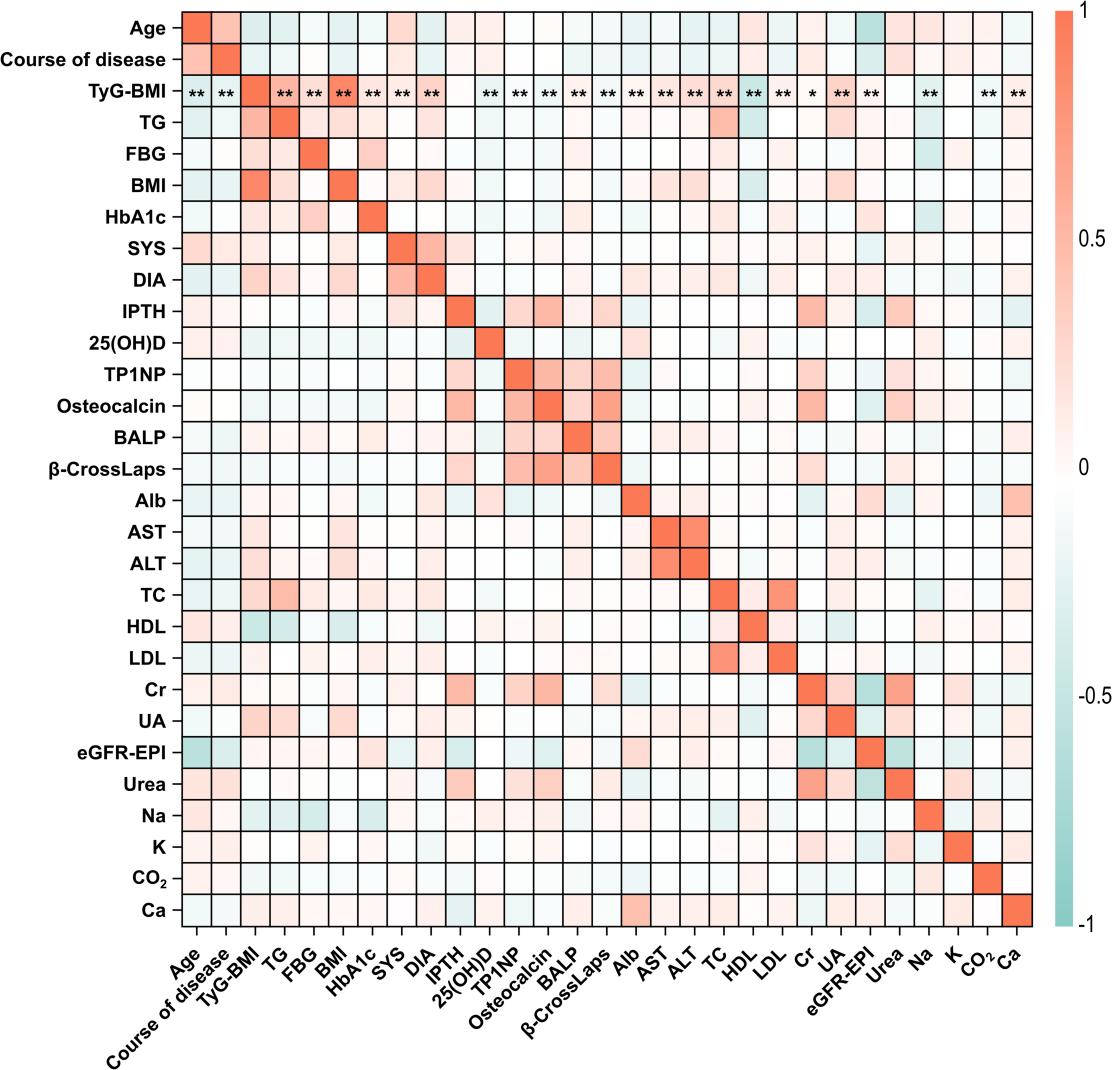
Correlation heatmap of clinical characteristics in T2D patients. The correlation coefficients between all the 29 clinical characteristics of T2D patients in this study were analyzed and illustrated. Correlation coefficients range from -1 to 1, with red indicating positive correlations and blue indicating negative correlations. The intensity of the color corresponds to the strength of the correlation. Specifically, the statistical significance of correlations between TyG-BMI and other characteristics were marked by asterisks. *P < 0.05, **P < 0.01. The full names for abbreviations of clinical characteristics are: TyG-BMI, triglyceride glucose-body mass index; TG, Triglycerides; FBG, Fasting blood glucose; BMI, Body mass index; SYS, Systolic pressure; DIA, Diastolic pressure; IPTH, Intact parathyroid hormone; 25(OH)D, 25-hydroxyvitamin D; TP1NP, Total procollagen type 1 N-terminal propeptide; BALP, Bone alkaline phosphatase; Alb, Albumin; AST, Aspartate aminotransferase; ALT, Alanine aminotransferase; TC, Total cholesterol; HDL, High density lipoprotein; LDL, Low density lipoprotein; Cr, Creatinine; UA, Uric acid; eGFR-EPI, Estimating glomerular filtration rate using the chronic kidney disease epidemiology collaboration (CKD-EPI) equation; Na, sodium; K, potassium; Ca, calcium.

To elucidate the associations among different clinical characteristics in T2D patients, we conducted a comprehensive correlation analysis among these indicators. Results showed that there are significant correlations among different clinical characteristics in T2D patients in this study (**Figure 2**; **Supplementary Table S2**). Among them, age was positively correlated with course of disease (*r* = 0.45, *P* < 0.01), but negatively correlated with eGFR-EPI (*r* = -0.54, *P* < 0.01). Creatinine (Cr) had significant correlations with many other characteristics including IPTH (*r* = 0.51, *P* < 0.01), TP1NP (*r* = 0.32, *P* < 0.01), osteocalcin (*r* = 0.53, *P* < 0.01), eGFR-EPI (*r* = -0.57, *P* < 0.01), and urea (*r* = 0.69, *P* < 0.01), indicating the potential role of Cr for metabolic ability in T2D patients. In addition, strong correlations were observed among other clinical characteristics. For instance, IPTH was significantly positively correlated with urea (*r* = 0.39, *P* < 0.01). FBG (fasting blood glucose) was significantly negatively correlated with sodium (*r* = 0.34, *P* < 0.01). Collectively, these correlations provide valuable insights into the interrelationships among different clinical and biochemical parameters and their potential impact on T2D progression.

### 3.3 Correlation analysis of TyG-BMI with sex, age, and course of disease

**Figure 3:**
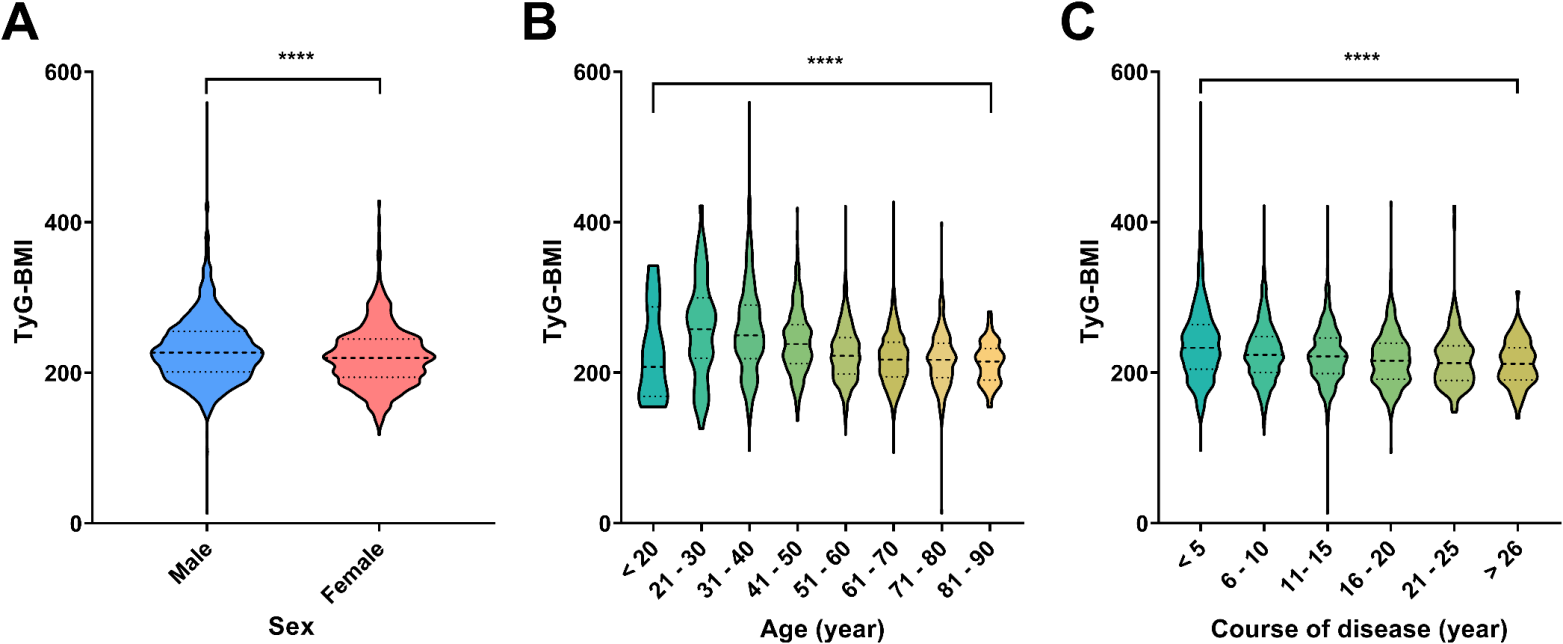
Distribution of TyG-BMI across different demographic and clinical categories. **(A)** Violin plots showed the distribution of TyG-BMI values by sex. Males and females are compared, with a significant difference indicated by ****P < 0.0001. **(B)** Violin plots showed the distribution of TyG-BMI across different age groups. The age groups are categorized as <20, 21-30, 31-40, 41-50, 51-60, 61-70, 71-80, and 81–90 years. A significant difference across age groups is marked by ****P < 0.0001. **(C)** Violin plots showed the distribution of TyG-BMI based on the course of disease in years. The disease course is divided into categories: <5, 6-10, 11-15, 16-20, 21-25, and >26 years. A significant difference in TyG-BMI with disease progression is marked by by ****P < 0.0001.

To further demonstrate the potential of TyG-BMI as a key indicator for the diagnosis of T2D, we assessed correlations between TyG-BMI and basic clinical information of patients. Interestingly, we found that TyG-BMI had relatively strong correlations with sex, age and course of disease (**Figure 3**). Specifically, we revealed that males exhibit a higher median TyG-BMI value compared to females, with a more pronounced distribution (*P* < 0.0001), suggesting a strong correlation between sex and TyG-BMI levels. While for different age groups, a significant decrease in TyG-BMI is observed with advancing age, except for age group of < 20. The lowest median TyG-BMI is observed in the 81–90 age group. The differences in TyG-BMI across age groups are statistically significant (*P* < 0.0001), indicating a strong negative correlation between age and TyG-BMI. For course of disease, a progressive decrease in TyG-BMI is evident as the disease course extends, with the lowest median TyG-BMI observed in the group with a disease course of over 26 years. The statistical analysis confirms a significant correlation between the course of disease and TyG-BMI, with a *P*-value of less than 0.0001. Taken together, the above correlation analysis reveals significant associations between TyG-BMI and sex, age, and the course of disease. Males, younger individuals, and those with a shorter disease course tend to have higher TyG-BMI values, underscoring the potential role of these factors in the pathophysiology of metabolic disorders.

### 3.4 Correlations between TyG-BMI and key characteristics of cardiovascular, renal, hepatic and bone biomarkers

**Figure 4:**
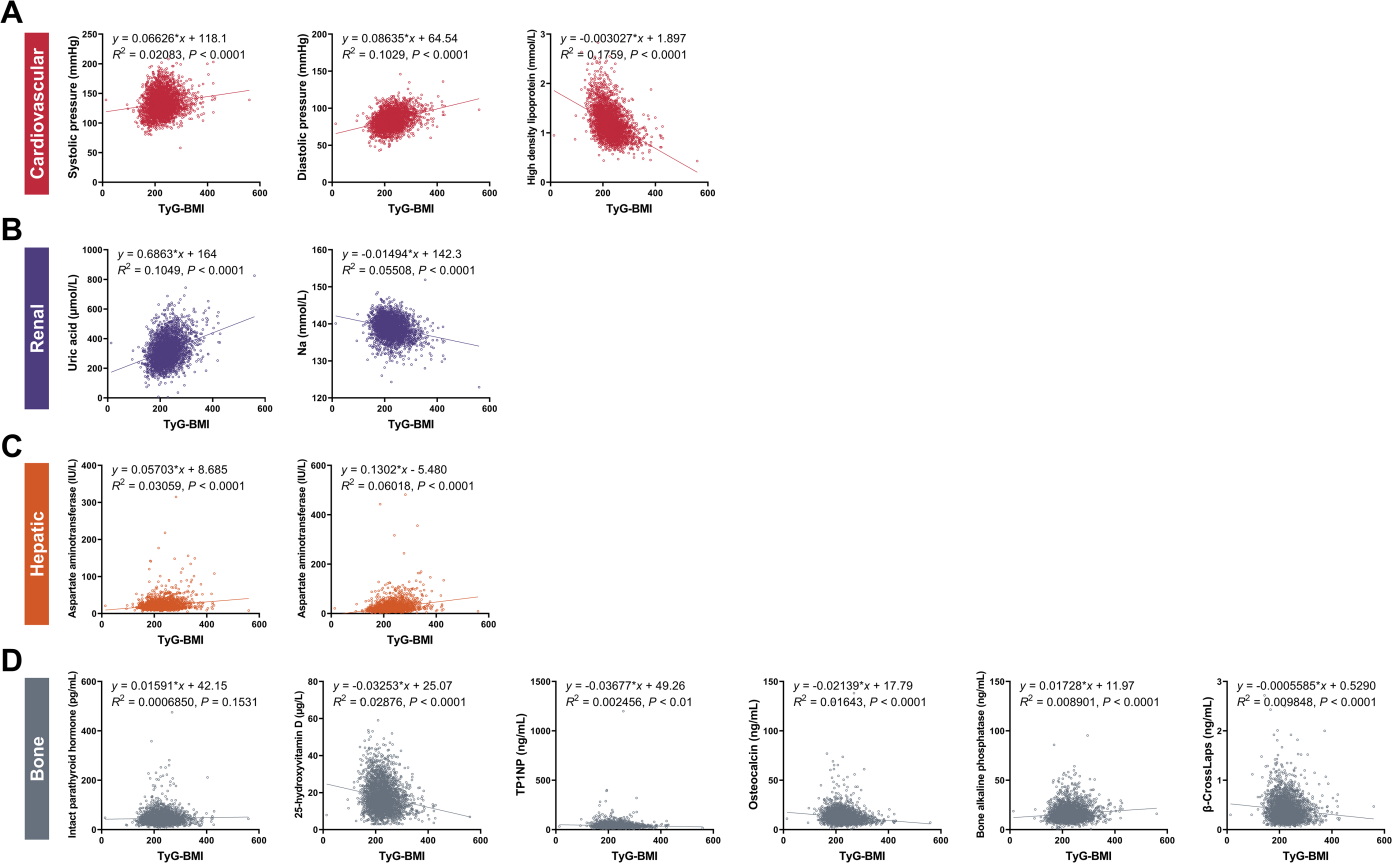
Correlations between TyG-BMI and key clinical characteristics of cardiovascular, renal, hepatic and bone biomarkers. **(A)** Cardiovascular biomarkers. Scatter plots showed the correlation between TyG-BMI and systolic pressure, diastolic pressure, and high-density lipoprotein (HDL). **(B)** Renal biomarkers. Scatter plots showed the correlation between TyG-BMI and uric acid and creatinine levels. **(C)** Hepatic biomarkers. Scatter plots showed the correlation between TyG-BMI and aspartate aminotransferase (AST) and alanine aminotransferase (ALT) levels. **(D)** Bone biomarkers. Scatter plots showed the correlation between TyG-BMI and intact parathyroid hormone, 25-hydroxyvitamin D, TP1NP, osteocalcin, bone alkaline phosphatase and β-CrossLaps. TP1NP, Total procollagen type 1 N-terminal propeptide.

We analyzed and evaluated the correlations between TyG-BMI and key characteristics within each category of complication risks in T2D patients (**Figure 4**). Given the significant difference of TyG-BMI between male and female T2D patients, we further analyzed the correlations in different sex subgroups (**Supplementary Figures S1, S2**).

For cardiovascular risk, our analysis revealed a significant association between elevated TyG-BMI and increased cardiovascular biomarkers. Specifically, patients with higher TyG-BMI exhibited higher systolic and diastolic blood pressures, indicative of a greater burden of hypertension. Correspondingly, these patients had reduced levels of high-density lipoprotein (HDL), often referred to as “good” cholesterol. Specifically, the systolic pressure showed a positive correlation with TyG-BMI (*y* = 0.06626*x* + 118.1, *R^2^* = 0.02083, *P* < 0.0001), indicating that higher TyG-BMI values are associated with increased systolic blood pressure. Similarly, diastolic pressure also demonstrated a positive correlation (*y* = 0.08635*x* + 64.54, *R^2^* = 0.1029, *P* < 0.0001). High-density lipoprotein levels showed a negative correlation with TyG-BMI (*y* = -0.003027*x* + 1.897, *R^2^* = 0.1759, *P* < 0.0001), suggesting that as TyG-BMI increases, HDL levels decrease, which is a risk factor for cardiovascular diseases. For sex subgroups, male patients showed higher correlations between TyG-BMI and diastolic blood pressure (*R^2^* = 0.1221) and HDL (*R^2^* = 0.1907), but lower correlations between TyG-BMI and systolic blood pressure (*R^2^* = 0.0233), compared with female patients.

For renal risks, we highlighted a concerning link between TyG-BMI and renal impairment. Patients with higher TyG-BMI values were more likely to have increased uric acid levels and reduced sodium (Na), both of which are known to adversely affect kidney function. This suggests that TyG-BMI could serve as a predictive marker for the development of diabetic nephropathy. Specifically, the uric acid levels were positively correlated with TyG-BMI (*y* = 0.6863*x* + 164, *R^2^* = 0.1049, *P* < 0.0001), indicating that higher TyG-BMI values are associated with increased uric acid levels, which can contribute to renal dysfunction. Conversely, sodium levels showed a negative correlation (*y* = -0.01494*x* + 142.3, *R^2^* = 0.05508, *P* < 0.0001), further supporting the association between TyG-BMI and impaired renal function. For sex subgroups, male patients showed higher correlations between TyG-BMI and uric acid (*R^2^* = 0.1084) and Na (*R^2^* = 0.06922) compared with female patients.

For hepatic risks, hepatic biomarkers were also found to be associated with TyG-BMI. Patients with elevated TyG-BMI showed increased levels of aspartate aminotransferase (AST) and alanine aminotransferase (ALT), enzymes that are indicative of liver damage. This association suggests the potential of TyG-BMI as a non-invasive tool for monitoring liver health in T2D patients. Specifically, AST levels were positively correlated with TyG-BMI (*y* = 0.05703*x* + 8.685, *R^2^* = 0.03059, *P* < 0.0001), suggesting that higher TyG-BMI values are linked to increased AST levels, indicative of liver stress or damage. ALT levels also demonstrated a positive correlation (*y* = 0.1302*x* - 5.480, *R^2^* = 0.06018, *P* < 0.0001), reinforcing the association between TyG-BMI and hepatic dysfunction. For sex subgroups, male patients showed higher correlations between TyG-BMI and AST (*R^2^* = 0.03215) and ALT (*R^2^* = 0.06079) compared with female patients.

For bone risks, our findings indicated that TyG-BMI is correlated with bone health. Patients with higher TyG-BMI had lower levels of 25-hydroxyvitamin D, TP1NP, osteocalcin and β-CrossLaps and higher levels of IPTH and bone alkaline phosphatase, which are markers of bone metabolism and turnover. This suggests that TyG-BMI may be a useful indicator for assessing the risk of osteoporosis and other bone-related complications in T2D. Specifically, IPTH (*y* = 0.01591*x* + 42.15, *R^2^* = 0.0006850, *P* < 0.0001) and bone alkaline phosphatase (*y* = 0.01728*x* + 11.97, *R^2^* = 0.008901, *P* < 0.0001) both showed positive correlations with TyG-BMI, indicating that higher TyG-BMI values are associated with increased IPTH and bone alkaline phosphatase levels, which can lead to abnormal mineral absorption and bone turnover. On the contrary, 25-hydroxyvitamin D levels were negatively correlated with TyG-BMI (*y* = -0.03253*x* + 25.07, *R^2^* = 0.02876, *P* < 0.0001), suggesting a potential impact on bone resorption. TP1NP levels were also negatively correlated with TyG-BMI (*y* = -0.03677*x* + 49.26, *R^2^* = 0.002456, *P* < 0.01), indicating the association between TyG-BMI and reduced bone formation as well as decreased osteoblast activity. Osteocalcin showed a negative correlation with TyG-BMI (*y* = -0.02139*x* + 17.79, *R^2^* = 0.01643, *P* < 0.0001), indicating that higher TyG-BMI values are associated with lower osteocalcin levels, which can affect bone formation. β-CrossLaps levels were negatively correlated with TyG-BMI (*y* = -0.0005585*x* + 0.5290, *R^2^* = 0.009848, *P* < 0.0001), further supporting the association between TyG-BMI and reduced bone resorption. For sex subgroups, female patients showed higher correlations between TyG-BMI and IPTH (*R^2^* = 0.006124), TP1NP (*R^2^* = 0.005047), osteocalcin (*R^2^* = 0.01759), and β-CrossLaps (*R^2^* = 0.02625), but lower correlations between TyG-BMI and 25-hydroxyvitamin D (*R^2^* = 0.02766) and bone alkaline phosphatase (*R^2^* = 0.008257), compared with male patients.

In conclusion, the above results indicate that higher TyG-BMI values are significantly associated with increased trends of cardiovascular, renal, hepatic, and bone biomarkers in T2D patients, as evidenced by the strong correlations with different biomarkers of these conditions. These findings emphasize the importance of integrating TyG-BMI into clinical practice for comprehensive risk assessment and management in T2D. Further research is needed to explore the underlying mechanisms and to validate the clinical utility of TyG-BMI in diverse populations and sex subgroups.

### 3.5 Cardiovascular characteristics of TyG-BMI subgroup in T2D patients

**Figure 5:**
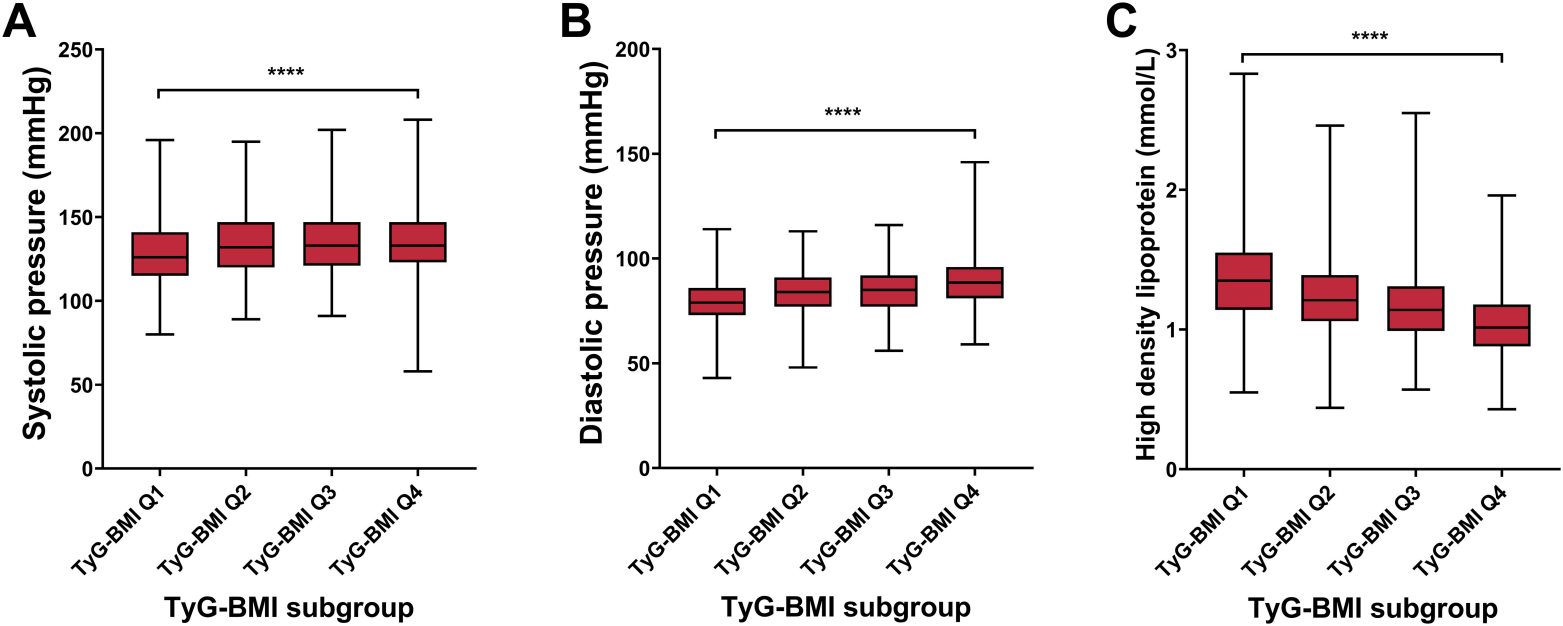
Cardiovascular biomarkers of TyG-BMI subgroup in T2D patients. Box plots showed the distribution of systolic pressure, diastolic pressure, and high-density lipoprotein (HDL) levels across quartiles of TyG-BMI. **(A)** Systolic pressure. The box plot showed the median, interquartile range, and outliers of systolic pressure measurements for each TyG-BMI quartile (Q1 to Q4). A significant increase in systolic pressure is observed with higher TyG-BMI quartiles. ****P < 0.0001. **(B)** Diastolic pressure. The box plot showed the median, interquartile range, and outliers of diastolic pressure measurements for each TyG-BMI quartile (Q1 to Q4). A significant increase in diastolic pressure is observed with higher TyG-BMI quartiles. ****P < 0.0001. **(C)** HDL. The box plot showed the median, interquartile range, and outliers of HDL measurements for each TyG-BMI quartile (Q1 to Q4). A significant decrease in HDL is observed with higher TyG-BMI quartiles. ****P < 0.0001.

To further validate the predictive ability of TyG-BMI for T2D complications, we analyzed the key cardiovascular risk characteristics of different TyG-BMI using quartile grouping (**Figure 5**; **Table 2**). For systolic pressure, we observed a significant increase in systolic pressure as TyG-BMI quartiles increase, with the highest quartile (TyG-BMI Q4) showing the most substantial elevation, marked by a *P*-value of less than 0.0001. Similar to systolic pressure, diastolic pressure also increases with higher TyG-BMI quartiles, with TyG-BMI Q4 again exhibiting the highest values, and the difference is statistically significant (*P* < 0.0001). In contrast to blood pressure, HDL levels decrease as TyG-BMI quartiles increase, with the lowest levels observed in TyG-BMI Q4, indicating a significant inverse relationship (*P* < 0.0001). Collectively, these findings further confirm that higher TyG-BMI is associated with increased blood pressure and decreased HDL levels, which would lead to increased risks of cardiovascular implications in T2D patients. For sex subgroups, female patients exhibited higher levels of systolic pressure and HDL, as well as milder rise in diastolic blood pressure, compared with male patients (**Supplementary Figure S3, S4**; **Supplementary Tables S3, S4**).

**Table 2:** Characteristics of age and bone, cardiovascular, renal and hepatic biomarkers of T2D patients in different TyG-BMI categories.

Characteristic	Q1, N = 745	Q2, N = 745	Q3, N = 745	Q4, N = 746	p-value
Age					<0.0001
Mean ± SD	60 ± 12	61 ± 11	59 ± 12	52 ± 13	
Median (IQR)	62 (56, 68)	61 (54, 68)	60 (51, 67)	53 (43, 62)	
Range	14, 87	14, 89	15, 90	15, 85	
Bone biomarker					
Intact parathyroid hormone (pg/mL)					0.3291
Mean ± SD	45 ± 24	45 ± 23	46 ± 25	47 ± 30	
Median (IQR)	40 (32, 51)	40 (32, 52)	41 (32, 54)	42 (31, 55)	
Range	10, 358	5, 231	6, 253	11, 476	
25-hydroxyvitamin D (µg/L)					<0.0001
Mean ± SD	19 ± 9	18 ± 8	18 ± 8	16 ± 7	
Median (IQR)	17 (13, 24)	17 (12, 23)	17 (12, 22)	15 (10, 20)	
Range	3, 70	3, 70	3, 67	3, 52	
TP1NP (ng/mL)					0.0170
Mean ± SD	44 ± 31	41 ± 20	39 ± 16	40 ± 47	
Median (IQR)	37 (29, 50)	36 (28, 49)	36 (27, 47)	35 (27, 45)	
Range	6, 400	11, 199	12, 118	6, 1,200	
Osteocalcin (ng/mL)					<0.0001
Mean ± SD	14.1 ± 8.4	13.2 ± 6.2	12.6 ± 5.8	11.8 ± 7.1	
Median (IQR)	12.4 (9.9, 16.1)	12.1 (9.3, 15.9)	11.5 (9.1, 15.0)	10.5 (8.4, 13.6)	
Range	1.4, 129.4	2.6, 65.4	2.5, 73.7	0.6, 138.2	
Bone alkaline phosphatase (ng/mL)					<0.0001
Mean ± SD	15 ± 7	16 ± 8	16 ± 7	17 ± 8	
Median (IQR)	13 (11, 18)	14 (11, 19)	14 (11, 19)	15 (11, 20)	
Range	1, 86	3, 58	3, 64	1, 95	
β-CrossLaps (ng/mL)					<0.0001
Mean ± SD	0.45 ± 0.28	0.40 ± 0.23	0.39 ± 0.20	0.37 ± 0.22	
Median (IQR)	0.39 (0.27, 0.55)	0.35 (0.25, 0.49)	0.34 (0.25, 0.48)	0.32 (0.22, 0.46)	
Range	0.08, 2.72	0.07, 2.50	0.06, 1.92	0.07, 2.00	
Cardiovascular biomarker					
Systolic pressure (mmHg)					<0.0001
Mean ± SD	129 ± 19	134 ± 19	135 ± 19	135 ± 19	
Median (IQR)	126 (115, 141)	132 (120, 147)	133 (121, 147)	133 (123, 147)	
Range	80, 196	89, 195	91, 202	58, 208	
Diastolic pressure (mmHg)					<0.0001
Mean ± SD	79 ± 10	84 ± 10	85 ± 11	89 ± 12	
Median (IQR)	79 (73, 86)	84 (77, 91)	85 (77, 92)	89 (81, 96)	
Range	43, 114	48, 113	56, 116	59, 146	
High density lipoprotein (mmol/L)					<0.0001
Mean ± SD	1.38 ± 0.34	1.24 ± 0.27	1.17 ± 0.26	1.05 ± 0.24	
Median (IQR)	1.35 (1.14, 1.55)	1.21 (1.06, 1.39)	1.14 (0.99, 1.31)	1.02 (0.88, 1.18)	
Range	0.55, 2.83	0.44, 2.46	0.57, 2.55	0.43, 1.96	
Renal biomarker					
Uric acid (µmol/L)					<0.0001
Mean ± SD	288 ± 78	309 ± 79	327 ± 87	358 ± 96	
Median (IQR)	283 (234, 334)	302 (258, 357)	317 (268, 378)	349 (292, 413)	
Range	7, 630	99, 593	5, 688	36, 825	
Na (mmol/L)					<0.0001
Mean ± SD	139.60 ± 2.65	139.17 ± 2.48	138.96 ± 2.50	138.06 ± 2.81	
Median (IQR)	139.70 (138.00, 141.20)	139.30 (137.60, 140.80)	139.00 (137.60, 140.70)	138.10 (136.20, 140.00)	
Range	126.00, 148.50	128.20, 147.10	124.30, 146.50	122.90, 151.90	
Hepatic biomarker					
Aspartate aminotransferase (IU/L)					<0.0001
Mean ± SD	20 ± 10	20 ± 10	21 ± 11	25 ± 20	
Median (IQR)	18 (15, 22)	18 (15, 22)	19 (15, 23)	20 (16, 28)	
Range	6, 142	7, 177	7, 218	5, 315	
Alanine aminotransferase (IU/L)					<0.0001
Mean ± SD	20 ± 20	21 ± 13	23 ± 18	32 ± 32	
Median (IQR)	17 (12, 22)	18 (13, 25)	19 (14, 27)	25 (16, 36)	
Range	1, 443	4, 125	5, 317	3, 482	

The full names of abbreviations are: TP1NP, Total procollagen type 1 N-terminal propeptide; Na, sodium.

### 3.6 Renal characteristics of TyG-BMI subgroup in T2D patients

**Figure 6:**
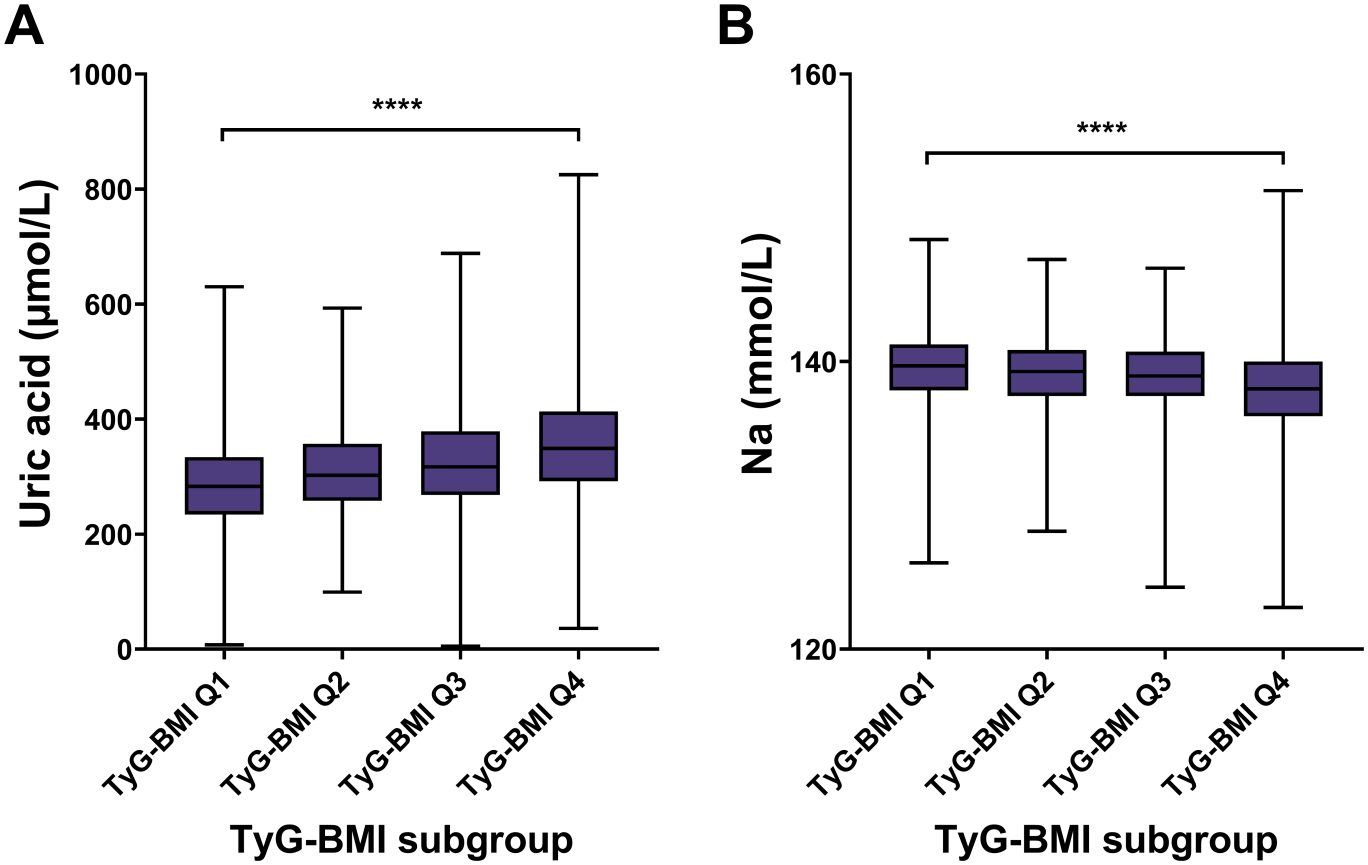
Renal biomarkers of TyG-BMI subgroup in T2D patients. Box plots showed the distribution of uric acid and sodium (Na) levels across quartiles of TyG-BMI. **(A)** Uric acid. The box plot showed the median, interquartile range, and outliers of uric acid measurements for each TyG-BMI quartile (Q1 to Q4). A significant increase in uric acid is observed with higher TyG-BMI quartiles. ****P < 0.0001. **(B)** Na. The box plot showed the median, interquartile range, and outliers of Na measurements for each TyG-BMI quartile (Q1 to Q4). A significant decrease in Na is observed with higher TyG-BMI quartiles. ****P < 0.0001.

For renal risk characteristics, we found a significant difference in uric acid levels across different TyG-BMI groups, with TyG-BMI Q1 having the lowest median uric acid level and TyG-BMI Q4 having the highest (*P* < 0.0001) (**Figure 6**; **Table 2**). Conversely, sodium levels exhibit a significant decrease from TyG-BMI Q1 to TyG-BMI Q4 (*P* < 0.0001). The median sodium level in TyG-BMI Q1 is higher compared to the other groups, with a progressive decrease observed in subsequent quartiles. The results suggest strong correlation between the TyG-BMI quartiles and uric acid and sodium levels. As the TyG-BMI quartile increases, there is a corresponding increase/decrease in the median levels of uric acid/sodium. This trend indicates that higher TyG-BMI values may be associated with elevated levels of uric acid and reduced levels of sodium, which could have implications for renal metabolic health and disease risk assessment. For sex subgroups, female patients showed milder trends (increase for uric acid and decrease for Na) compared with male patients (**Supplementary Figures S5, S6**; **Supplementary Tables S3, S4**).

### 3.7 Hepatic characteristics of TyG-BMI subgroup in T2D patients

**Figure 7:**
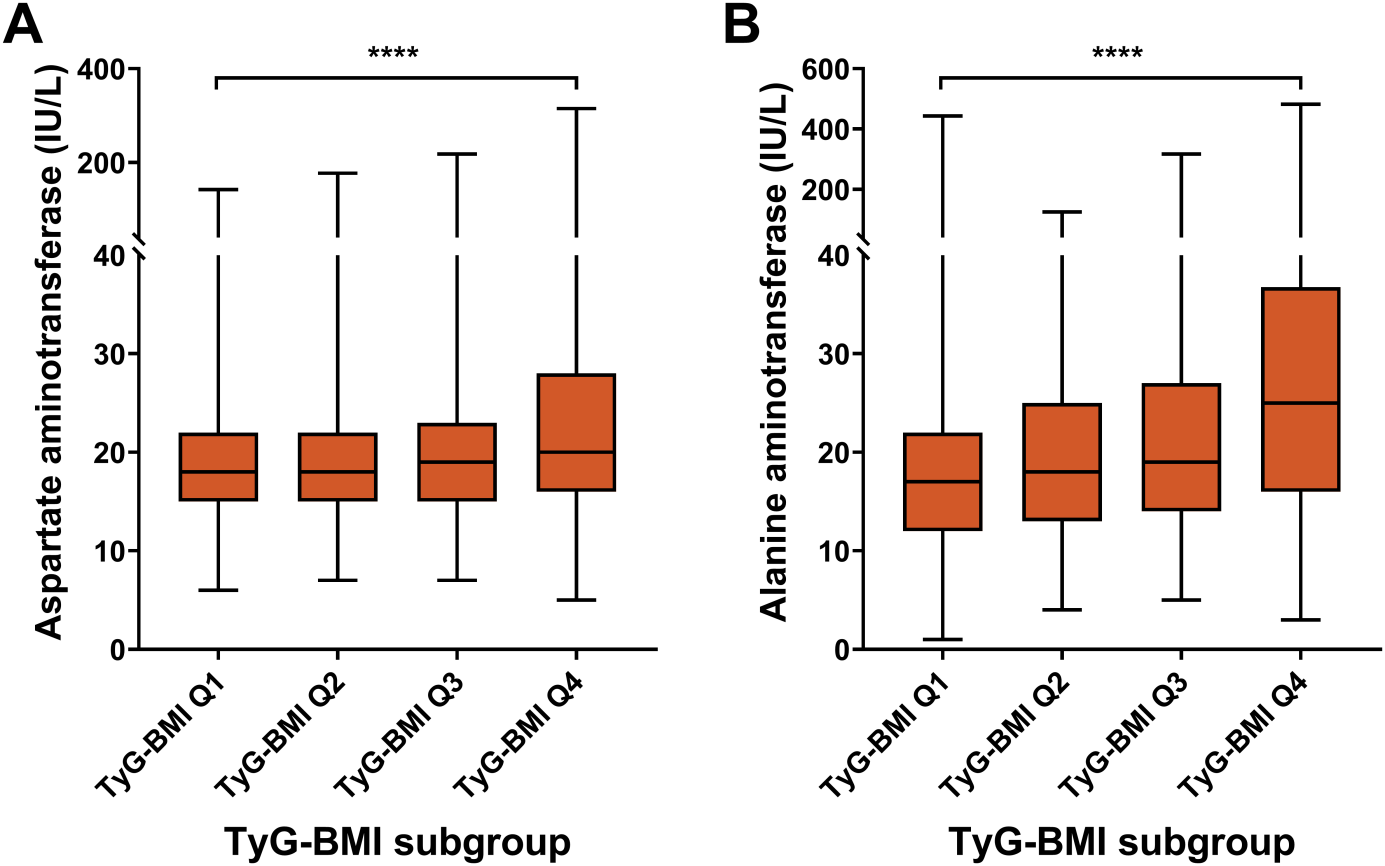
Hepatic biomarkers of TyG-BMI subgroup in T2D patients. Box plots showed the distribution of aspartate aminotransferase (AST) and alanine aminotransferase (ALT) levels across quartiles of TyG-BMI. **(A)** AST. The box plot showed the median, interquartile range, and outliers of AST measurements for each TyG-BMI quartile (Q1 to Q4). A significant increase in AST is observed with higher TyG-BMI quartiles. ****P < 0.0001. **(B)** ALT. The box plot showed the median, interquartile range, and outliers of ALT measurements for each TyG-BMI quartile (Q1 to Q4). A significant increase in ALT is observed with higher TyG-BMI quartiles. ****P < 0.0001.

For hepatic risk, the aspartate aminotransferase (AST) level had an increasing trend across TyG-BMI Q1 to Q4 subgroups (*P* < 0.0001) (**Figure 7**; **Table 2**). Similar to AST, ALT levels significantly increased from TyG-BMI Q1 to Q4, and reached the highest level in TyG-BMI Q4 subgroup (*P* < 0.0001). Taken together, these results indicate that both aspartate and alanine aminotransferase levels are significantly elevated with increasing TyG-BMI. This finding further suggests that the metabolic processes related to these enzymes may be altered in T2D patients, potentially due to differences in body mass index or other physiological factors. Further investigation is needed to explore the underlying mechanisms and the implications of these enzymatic changes for hepatic health. For sex subgroups, female patients showed milder increases for AST and ALT compared with male patients (**Supplementary Figures S7, S8**; **Supplementary Tables S3, S4**).

### 3.8 Bone characteristics of TyG-BMI subgroup in T2D patients

**Figure 8:**
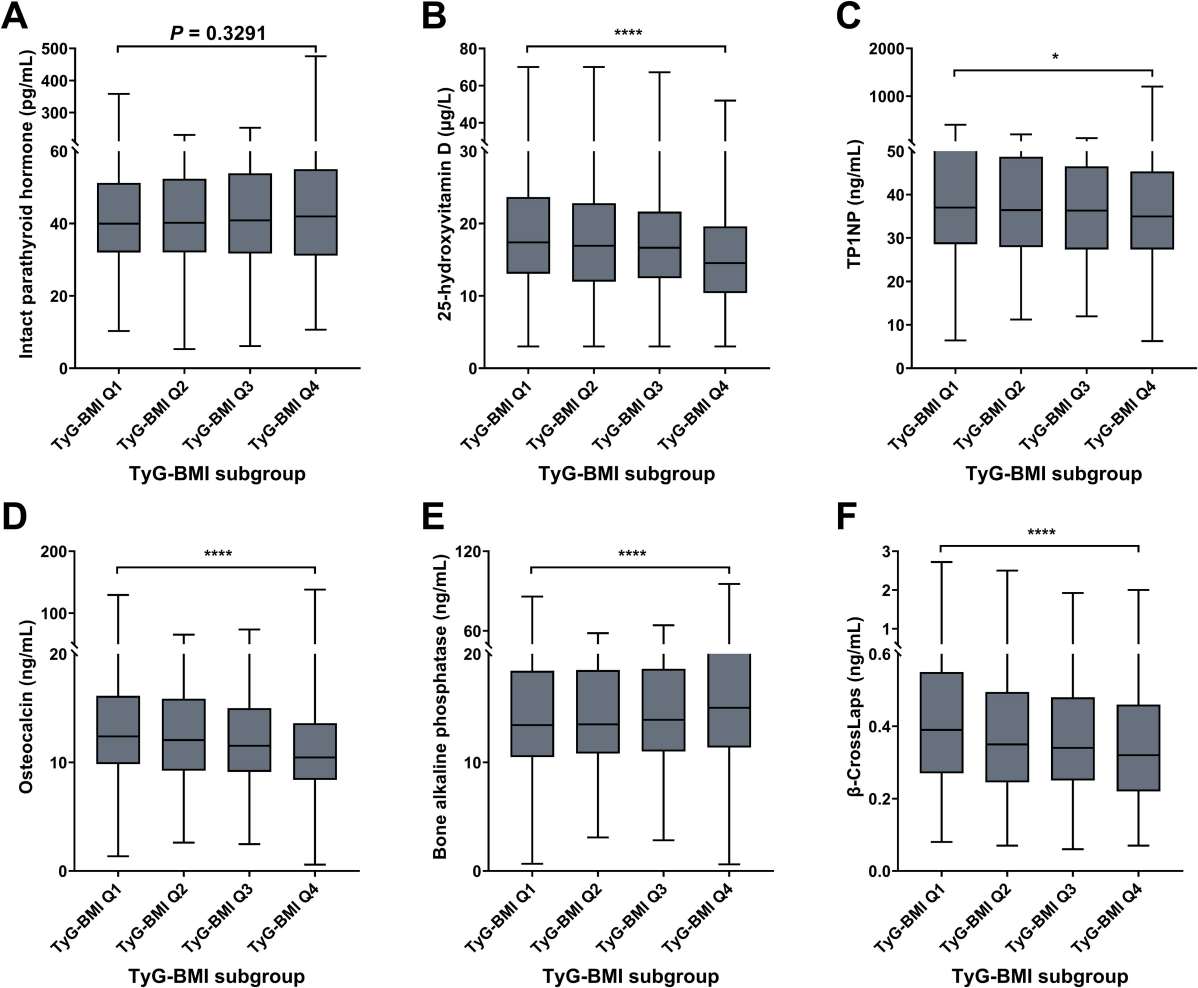
Bone biomarkers of TyG-BMI subgroup in T2D patients. Box plots showed the distribution of intact parathyroid hormone (IPTH), 25-hydroxyvitamin (25[OH]D), total procollagen type 1 N-terminal propeptide (TP1NP), osteocalcin; bone alkaline phosphatase (BALP) and β-CrossLaps levels across quartiles of TyG-BMI. **(A)** IPTH. The box plot showed the median, interquartile range, and outliers of IPTH measurements for each TyG-BMI quartile (Q1 to Q4). An increase in IPTH is observed with higher TyG-BMI quartiles. *P* = 0.3291. **(B)** 25(OH)D. The box plot showed the median, interquartile range, and outliers of 25(OH)D measurements for each TyG-BMI quartile (Q1 to Q4). A significant decrease in 25(OH)D is observed with higher TyG-BMI quartiles. ****P < 0.0001. **(C)** TP1NP. The box plot showed the median, interquartile range, and outliers of TP1NP measurements for each TyG-BMI quartile (Q1 to Q4). A significant decrease in TP1NP is observed with higher TyG-BMI quartiles. ****P < 0.0001. **(D)** Osteocalcin. The box plot showed the median, interquartile range, and outliers of osteocalcin measurements for each TyG-BMI quartile (Q1 to Q4). A significant decrease in osteocalcin is observed with higher TyG-BMI quartiles. ****P < 0.0001. **(E)** BALP. The box plot showed the median, interquartile range, and outliers of BALP measurements for each TyG-BMI quartile (Q1 to Q4). A significant increase in BALP is observed with higher TyG-BMI quartiles. ****P < 0.0001. **(F)** β-CrossLaps. The box plot showed the median, interquartile range, and outliers of β-CrossLaps measurements for each TyG-BMI quartile (Q1 to Q4). A significant decrease in β-CrossLaps is observed with higher TyG-BMI quartiles. ****P < 0.0001.

For bone risk characteristics, four indicators (25-hydroxyvitamin D, TP1NP, osteocalcin, and β-Crosslaps) decreased from TyG-BMI Q1 to Q4 subgroup, while two indicators (IPTH and bone alkaline phosphatase) exhibited an increasing trend (**Figure 8**; **Table 2**). For instance, olsteocalcin levels decrease across the TyG-BMI quartiles. The median levels are about 12.40 ng/mL in Q1, 12.06 ng/mL in Q2, 11.54 ng/mL in Q3, and 10.45 ng/mL in Q4, with highly significant differences between the quartiles (*P* < 0.0001). On the contrary, bone alkaline phosphatase levels show a converse trend, significantly increasing from TyG-BMI Q1 to Q4 subgroup (*P* < 0.0001). The median levels are around 13.43 ng/mL in Q1, 13.51 ng/mL in Q2, 13.92 ng/mL in Q3, and 15.04 ng/mL in Q4. In summary, these results demonstrate a clear association between increasing TyG-BMI quartiles and changes in different biochemical parameters related to bone health and vitamin D status, suggesting that higher TyG-BMI values are associated with increased levels of IPTH and bone alkaline phosphatase and decreased levels of 25-hydroxyvitamin D, TP1NP, osteocalcin, and β-Crosslaps. These findings further highlight the potential role of TyG-BMI as a biomarker for assessing bone metabolic health and complication risks in T2D patient. For sex subgroups, female patients exhibited more rapid trends of IPTH (increase), TP1NP (decrease), osteocalcin (decrease) and β-CrossLaps (decrease), but milder trends of 25-hydroxyvitamin D (decrease), compared with male patients (**Supplementary Figures S9, S10**; **Supplementary Tables S3, S4**).

## 4 Discussion

In this study, our findings indicate that TyG-BMI is not only a reflection of metabolic status but also strongly correlated with adverse biomarkers across cardiovascular, renal, hepatic, and bone domains in T2D patients, serving as a potential indicator for assessing risks of these complications. Specifically, the significant correlations identified between elevated TyG-BMI and increased systolic and diastolic blood pressures support its potential as a predictor of cardiovascular risk (22, 23). This is particularly notable given that hypertension is a prevalent complication in T2D, contributing to increased morbidity and mortality (24). The association between TyG-BMI and renal biomarkers is especially concerning. Elevated levels of uric acid, which we found to be positively correlated with higher TyG-BMI values, are known to contribute to kidney damage and the progression of diabetic nephropathy. This suggests that TyG-BMI could serve as a useful screening tool for early detection of renal impairment, prompting timely interventions that could prevent or mitigate progression to end-stage renal disease. In the context of hepatic health, our results showing correlations between TyG-BMI and liver enzyme levels (AST and ALT) are particularly significant. Elevated levels of these enzymes are indicative of liver stress or injury and have been linked to non-alcoholic fatty liver disease (NAFLD), which is common among individuals with T2D. The ability of TyG-BMI to signal potential hepatic dysfunction could enhance monitoring strategies for patients, allowing for earlier identification of liver-related complications that can often go unnoticed until they are advanced. Collectively, these findings emphasize the critical role of TyG-BMI as a comprehensive biomarker that can aid clinicians in assessing and managing the multifaceted complications associated with T2D. Unlike other indicators (e.g., HOMA-IR), TyG-BMI requires only routine lipid/glucose tests and BMI, avoiding costly insulin assays. While HOMA-IR remains gold standard for IR, TyG-BMI offers comparable predictive value for complications (e.g., CVD) in resource-limited settings. By integrating TyG-BMI into routine clinical assessments, it is expected to improve risk stratification and develop more targeted interventions for this patient population in the future.

In particular, our findings regarding bone health are critical, as they reveal a potentially underrecognized aspect of T2D complications. The associations between TyG-BMI and bone metabolism markers suggest that higher TyG-BMI could correlate with an increased risk of osteoporosis and fractures. Specifically, our data reveal an apparent paradox in bone turnover markers: while elevated TyG-BMI correlated with increased bone alkaline phosphatase (BALP, a formation marker), it simultaneously associated with decreased osteocalcin (another formation marker) and β-CrossLaps (resorption marker). This inconsistency may reflect uncoupled bone remodeling in T2D pathophysiology. The mechanisms of insulin resistance can be proposed in three ways: (1) impairing osteoblast function via hyperglycemia-induced oxidative stress and AGE accumulation, reducing osteocalcin synthesis; (2) triggering compensatory osteoblast proliferation (elevating BALP) to counter increased apoptosis; and (3) suppressing osteoclast activity through adipokine dysregulation (e.g., elevated adiponectin), lowering β-CrossLaps. Additionally, osteocalcin itself regulates insulin sensitivity - creating a bidirectional feedback loop where insulin resistance suppresses osteocalcin, further exacerbating metabolic dysfunction. This decoupling of formation/resorption pathways may explain why diabetic bone disease features both low turnover (reduced biomarkers) and high fracture risk, distinct from classic osteoporosis. Given that bone health is often overlooked in the management of diabetes, the identification of TyG-BMI as a relevant indicator in this context may prompt further research into preventative strategies and management of bone health in T2D patients. Future studies should incorporate dual-energy X-ray absorptiometry (DXA) to measure bone mineral density (BMD), as discordance between bone turnover markers and actual bone density is well-documented in diabetes. This would clarify whether TyG-BMI-driven biomarker changes translate to clinically meaningful fracture risk.

Meanwhile, our stratified analyses identified clinically significant sex differences in the associations between the TyG-BMI index and organ-specific biomarkers. A key observation was the consistently elevated TyG-BMI values in males compared to females, consistent with established sexual dimorphism in adiposity distribution and insulin sensitivity (25, 26). Crucially, the strength and pattern of associations between TyG-BMI and specific biomarkers exhibited marked sex-specific variations. For cardiovascular and hepatic risks, TyG-BMI demonstrated stronger correlations in males for diastolic blood pressure (male: *R^2^* = 0.1221 *vs* female: *R^2^* = 0.0555) and ALT levels (male: *R^2^* = 0.06079 *vs* female: *R^2^* = 0.05366), suggesting a heightened male vulnerability to hypertension and steatohepatitis associated with insulin resistance and adiposity. While for bone metabolism, conversely, females exhibited stronger negative associations between TyG-BMI and bone turnover markers. Per unit increase in TyG-BMI, females showed steeper declines in osteocalcin (female *R^2^* = 0.01759 *vs* male 0.01159) and β-CrossLaps (female 0.02625 *vs* male 0.0002098), indicating a greater disruption of bone homeostasis in females. Several interconnected, sex-specific mechanisms are likely responsible for these divergent associations. Firstly, hormonal modulation plays a crucial role. In females with elevated TyG-BMI and type 2 diabetes (T2D), compromised estrogenic protection may accelerate uncoupled bone remodeling, whereas conversely, lower testosterone levels in metabolically dysfunctional males may exacerbate ectopic fat deposition, thereby potentiating liver injury. Beyond hormones, body composition differences significantly influence these pathways. The visceral adiposity component captured by TyG-BMI appears to drive hepatic insulin resistance more robustly in males, while gluteofemoral fat in females may confer some protection against dyslipidemia yet fails to mitigate TyG-BMI-associated bone loss. Furthermore, genetic factors contribute substantially. Sex chromosome-linked genes (e.g., polymorphisms in sex hormone-binding globulin [SHBG]) may differentially regulate triglyceride metabolism and vitamin D bioavailability between sexes. In addition, iatrogenic factors play important roles. Although recent medication users were excluded, historical exposure to therapies (e.g., androgen-deprivation therapy in males, menopausal hormone therapy in females) could induce lasting metabolic reprogramming influencing these relationships. Collectively, these pronounced sex divergences underscore critical clinical implications, highlighting the necessity for sex-tailored approaches. For instance, distinct TyG-BMI risk thresholds (e.g., >250 for males *vs >*230 for females) may be warranted to trigger specific screenings, such as hepatic function panels. Future mechanistic studies should directly investigate these causal pathways by incorporating concurrent measurements of sex hormones, precise body composition quantification (e.g., via DXA/VAT imaging), and relevant pharmacogenomic variants.

The clinical implications of our findings are multifaceted. First, TyG-BMI offers a pragmatic, low-cost tool for early identification of T2D patients at high risk for multi-organ complications. By integrating TyG-BMI into routine diabetes care (e.g., during annual screenings), clinicians could prioritize intensive monitoring (e.g., hepatic ultrasound for NAFLD, DEXA scans for osteoporosis) or targeted interventions (e.g., SGLT2 inhibitors for cardiorenal protection) for high-risk subgroups, optimizing resource allocation in resource-constrained settings. Second, TyG-BMI’s strong correlation with hepatic (ALT/AST) and bone turnover markers (e.g., osteocalcin, β-CrossLaps) highlights its potential as a surrogate endpoint in clinical trials evaluating novel therapies for diabetes-related extra-glycemic complications. Crucially, our data bridge a critical gap in diabetes management: bone health. The inverse relationship between TyG-BMI and 25(OH)D/osteocalcin underscores the need to include bone metabolism assessments in routine T2D metabolic evaluations, potentially reducing fracture-related morbidity. Meanwhile, it should be noted that interpretation of bone turnover associations must account for key confounders unmeasured in our study. For instance, widespread vitamin D deficiency (25(OH)D <20 µg/L in 68% of T2D patients) may independently suppress osteoblast activity and amplify PTH secretion - potentially obscuring TyG-BMI’s true relationship with bone formation markers like TP1NP and osteocalcin. Also, renal impairment (eGFR-EPI <60 mL/min in 22% of patients) may profoundly alter bone metabolism. Considering these, further efforts must include longitudinal vitamin D supplementation data, medication-adjusted models, and stratification by CKD stage to isolate TyG-BMI’s specific contribution to diabetic bone disease. Collectively, future studies should define TyG-BMI thresholds (e.g., Q4 >250) for complication risk and validate its additive value alongside emerging biomarkers (e.g., hs-CRP, FIB-4) to build personalized risk engines. Implementing TyG-BMI could transform T2D care from glucose-centric management to holistic multi-organ protection, ultimately reducing hospitalizations, disability, and healthcare costs.

In addition, there are several limitations warrant consideration when interpreting our findings. First, as a single-center, cross-sectional study, our design precludes causal inferences about TyG-BMI and complication development. The absence of longitudinal data means we cannot establish whether elevated TyG-BMI precedes or results from organ damage. Second, while we adjusted for key variables, unmeasured confounders may influence results, including lifestyle factors (e.g., dietary patterns, physical activity, smoking), socioeconomic determinants of health, and genetic predispositions. Finally, we defined complications using biomarker thresholds without accounting for race/sex-specific variations in normal ranges (e.g., ALT upper limits). Future multi-center cohorts including longitudinal designs tracking TyG-BMI trajectories and incident complications and comprehensive confounder adjustment using electronic health records should address these gaps.

In conclusion, in this study we first identified the strong correlations between TyG-BMI and cardiovascular, renal, hepatic, and bone biomarkers in T2D patients, highlighting its potential role as a novel indicator for assessing risks of these complications. Further studies are needed to explore its mechanistic pathways and validate its application across diverse clinical contexts. By embracing TyG-BMI as a standard measure in diabetes care, these efforts can enhance risk assessment and ultimately improve the life quality of T2D patients.

## Data Availability

The original contributions presented in the study are included in the article/**Supplementary Material**. Further inquiries can be directed to the corresponding author.
