# Supplementary material for: Associations between triglyceride glucose-body mass index and cardiovascular, renal, hepatic and bone biomarkers in patients with type 2 diabetes: a single-center, cross-sectional study
Source: Front Endocrinol (Lausanne). 2025 Sep 12;16:1566818. doi: 10.3389/fendo.2025.1566818 (PMC12463613; doi:10.3389/fendo.2025.1566818)
Supplement: Supplementary file 1 [file DataSheet1.docx]

**Supporting information for:**

Associations between triglyceride glucose-body mass index and cardiovascular, renal, hepatic and bone biomarkers in patients with type 2 diabetes: A single-center, cross-sectional study

Nan Xu^1,§^, Kunyi Wu^2,§^, Ting La^3^, Ruo Zhang^4^, Bo Cao^1,2,*^

^1^ Department of Clinical Laboratory, The Second Affiliated Hospital of Xi’an Jiaotong University, Xi’an, 710004, Shaanxi, China

^2^ Core Research Laboratory, The Second Affiliated Hospital of Xi’an Jiaotong University, Xi’an, 710004, Shaanxi, China

^3^ National-Local Joint Engineering Research Center of Biodiagnosis & Biotherapy, The Second Affiliated Hospital of Xi’an Jiaotong University, Xi’an, 710004, Shaanxi, China

^4^ Department of Endocrinology, The Second Affiliated Hospital of Xi’an Jiaotong University, Xi’an, 710004, Shaanxi, China

***Correspondence to**:

Bo Cao

Email: [bo_cao@xjtu.edu.cn](mailto:bo_cao@xjtu.edu.cn); [bocao@snnu.edu.cn](mailto:bocao@snnu.edu.cn)

^§^**Authorship note:**

Nan Xu and Kunyi Wu contributed equally to this work.

**This file contains:**

*Supplementary figure S1 to S10 (Figure S1 to S10)*

*Supplementary table S1, S3, S4 (Table S1, S3, S4)*


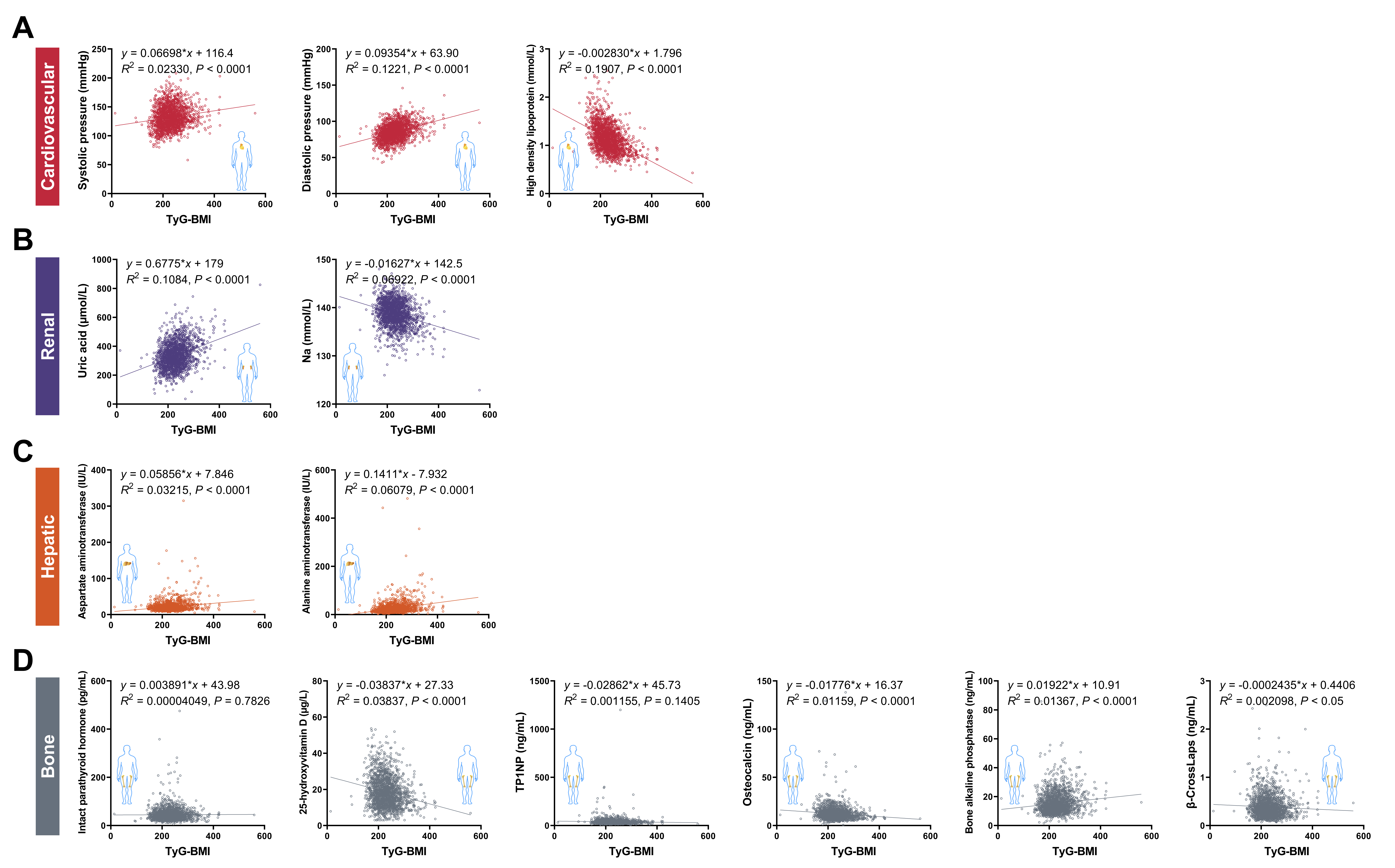


**Figure S1. Correlations between TyG-BMI and key clinical characteristics of cardiovascular, renal, hepatic and bone biomarkers in male T2D patients.**

(A) Cardiovascular biomarkers. Scatter plots showed the correlation between TyG-BMI and systolic pressure, diastolic pressure, and high-density lipoprotein (HDL). (B) Renal biomarkers. Scatter plots showed the correlation between TyG-BMI and uric acid and creatinine levels. (C) Hepatic biomarkers. Scatter plots showed the correlation between TyG-BMI and aspartate aminotransferase (AST) and alanine aminotransferase (ALT) levels. (D) Bone biomarkers. Scatter plots showed the correlation between TyG-BMI and intact parathyroid hormone, 25-hydroxyvitamin D, TP1NP, osteocalcin, bone alkaline phosphatase and β-CrossLaps. TP1NP - Total procollagen type 1 N-terminal propeptide.


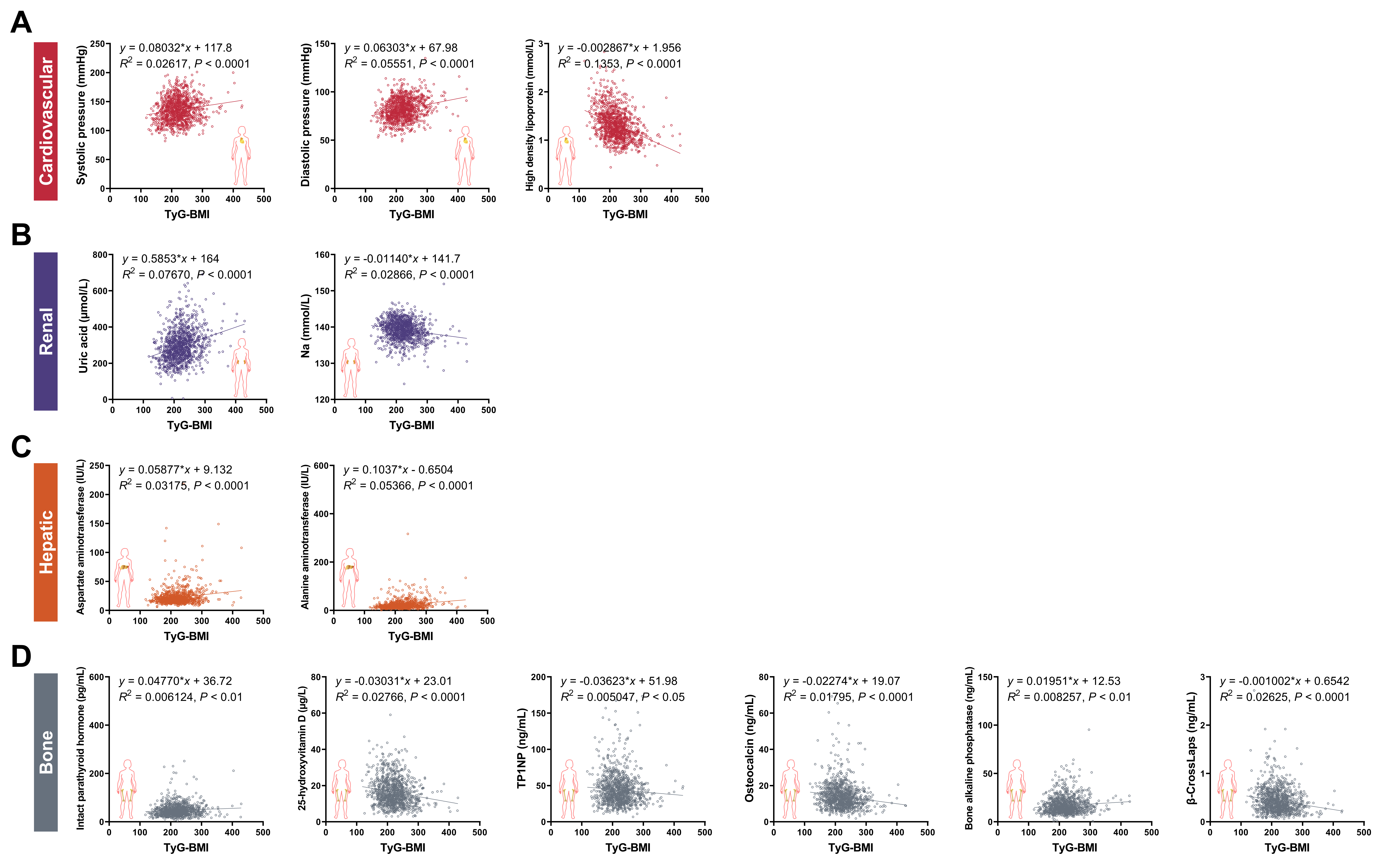


**Figure S2. Correlations between TyG-BMI and key clinical characteristics of cardiovascular, renal, hepatic and bone biomarkers in female T2D patients.**

(A) Cardiovascular biomarkers. Scatter plots showed the correlation between TyG-BMI and systolic pressure, diastolic pressure, and high-density lipoprotein (HDL). (B) Renal biomarkers. Scatter plots showed the correlation between TyG-BMI and uric acid and creatinine levels. (C) Hepatic biomarkers. Scatter plots showed the correlation between TyG-BMI and aspartate aminotransferase (AST) and alanine aminotransferase (ALT) levels. (D) Bone biomarkers. Scatter plots showed the correlation between TyG-BMI and intact parathyroid hormone, 25-hydroxyvitamin D, TP1NP, osteocalcin, bone alkaline phosphatase and β-CrossLaps. TP1NP - Total procollagen type 1 N-terminal propeptide.


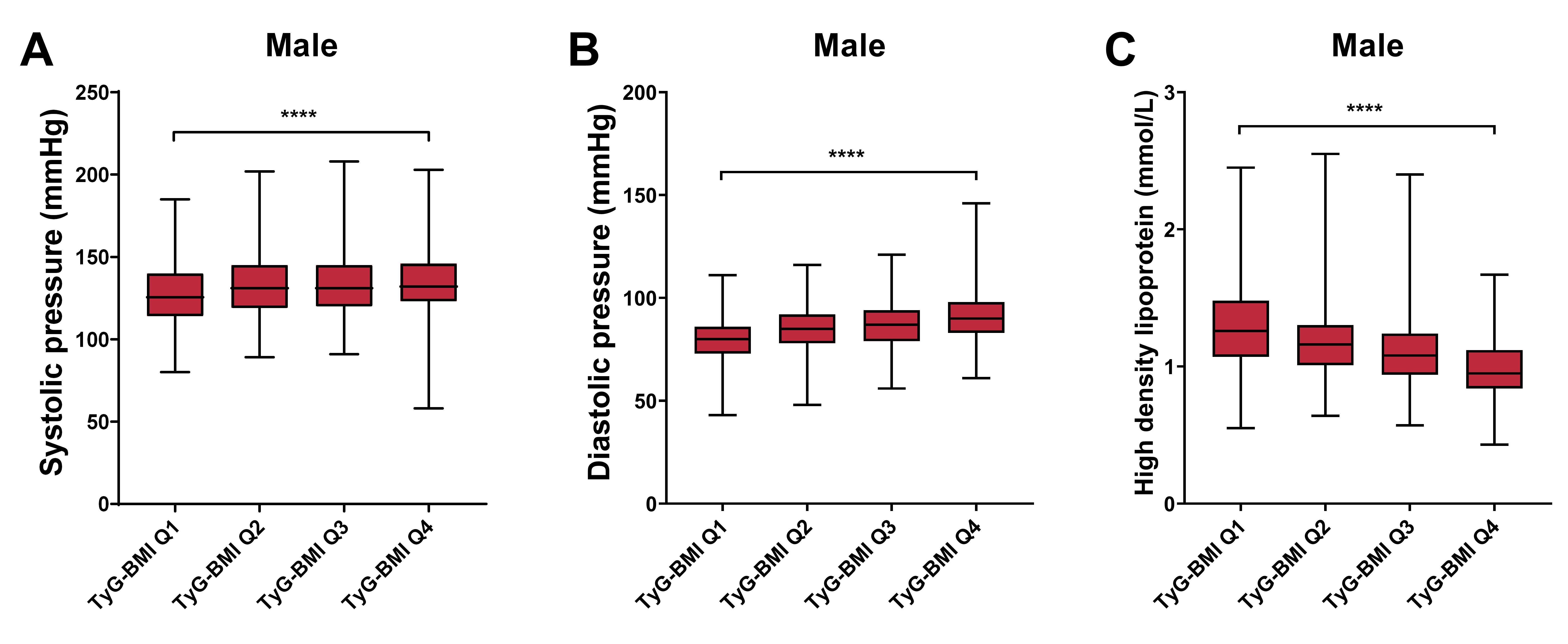


**Figure S3. Cardiovascular biomarkers of TyG-BMI subgroup in male T2D patients.**

Box plots showed the distribution of systolic pressure, diastolic pressure, and high-density lipoprotein (HDL) levels across quartiles of TyG-BMI. (A) Systolic pressure. The box plot showed the median, interquartile range, and outliers of systolic pressure measurements for each TyG-BMI quartile (Q1 to Q4). A significant increase in systolic pressure is observed with higher TyG-BMI quartiles. *****P* < 0.0001. (B) Diastolic pressure. The box plot showed the median, interquartile range, and outliers of diastolic pressure measurements for each TyG-BMI quartile (Q1 to Q4). A significant increase in diastolic pressure is observed with higher TyG-BMI quartiles. *****P* < 0.0001. (C) HDL. The box plot showed the median, interquartile range, and outliers of HDL measurements for each TyG-BMI quartile (Q1 to Q4). A significant decrease in HDL is observed with higher TyG-BMI quartiles. *****P* < 0.0001.


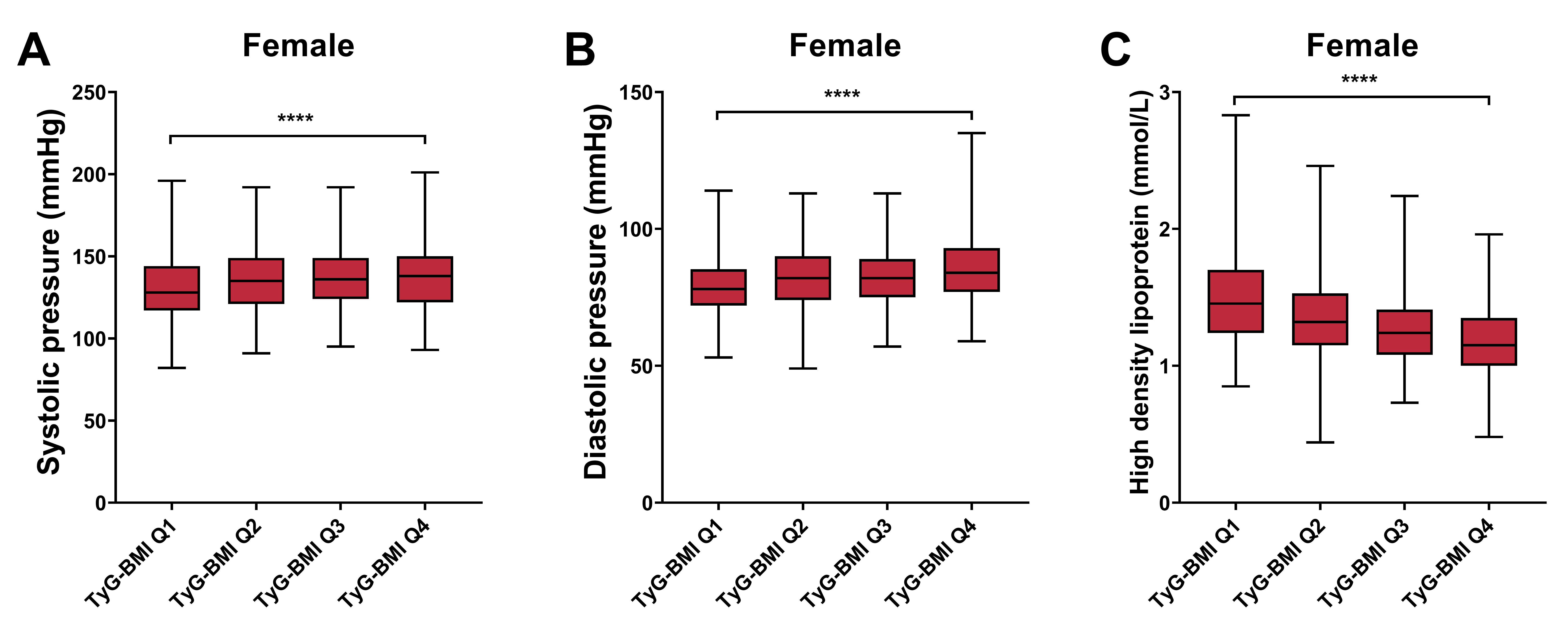


**Figure S4. Cardiovascular biomarkers of TyG-BMI subgroup in female T2D patients.**

Box plots showed the distribution of systolic pressure, diastolic pressure, and high-density lipoprotein (HDL) levels across quartiles of TyG-BMI. (A) Systolic pressure. The box plot showed the median, interquartile range, and outliers of systolic pressure measurements for each TyG-BMI quartile (Q1 to Q4). A significant increase in systolic pressure is observed with higher TyG-BMI quartiles. *****P* < 0.0001. (B) Diastolic pressure. The box plot showed the median, interquartile range, and outliers of diastolic pressure measurements for each TyG-BMI quartile (Q1 to Q4). A significant increase in diastolic pressure is observed with higher TyG-BMI quartiles. *****P* < 0.0001. (C) HDL. The box plot showed the median, interquartile range, and outliers of HDL measurements for each TyG-BMI quartile (Q1 to Q4). A significant decrease in HDL is observed with higher TyG-BMI quartiles. *****P* < 0.0001.


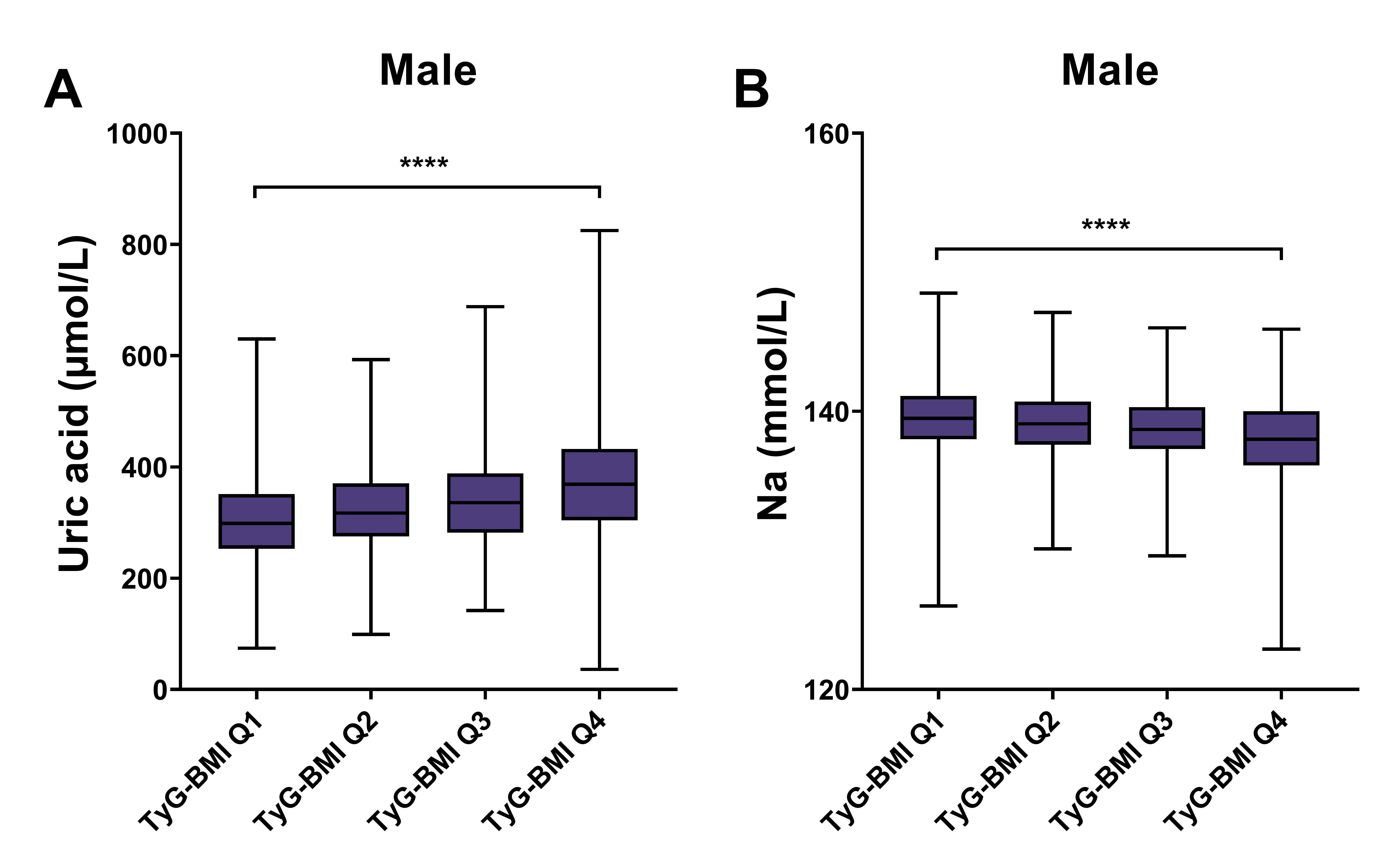


**Figure S5. Renal biomarkers of TyG-BMI subgroup in male T2D patients.**

Box plots showed the distribution of uric acid and sodium (Na) levels across quartiles of TyG-BMI. (A) Uric acid. The box plot showed the median, interquartile range, and outliers of uric acid measurements for each TyG-BMI quartile (Q1 to Q4). A significant increase in uric acid is observed with higher TyG-BMI quartiles. *****P* < 0.0001. (B) Na. The box plot showed the median, interquartile range, and outliers of Na measurements for each TyG-BMI quartile (Q1 to Q4). A significant decrease in Na is observed with higher TyG-BMI quartiles. *****P* < 0.0001.


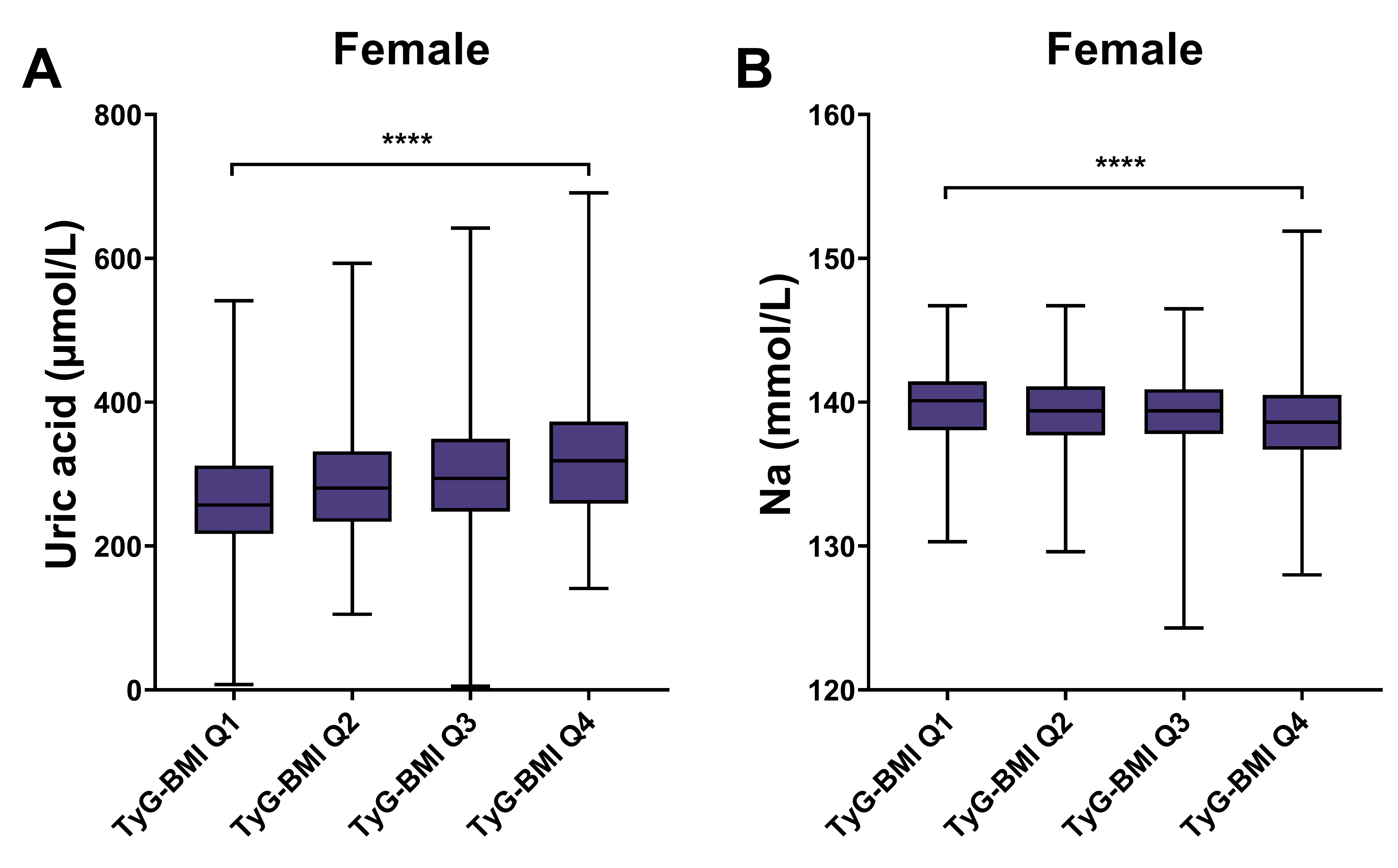


**Figure S6. Renal biomarkers of TyG-BMI subgroup in female T2D patients.**

Box plots showed the distribution of uric acid and sodium (Na) levels across quartiles of TyG-BMI. (A) Uric acid. The box plot showed the median, interquartile range, and outliers of uric acid measurements for each TyG-BMI quartile (Q1 to Q4). A significant increase in uric acid is observed with higher TyG-BMI quartiles. *****P* < 0.0001. (B) Na. The box plot showed the median, interquartile range, and outliers of Na measurements for each TyG-BMI quartile (Q1 to Q4). A significant decrease in Na is observed with higher TyG-BMI quartiles. *****P* < 0.0001.


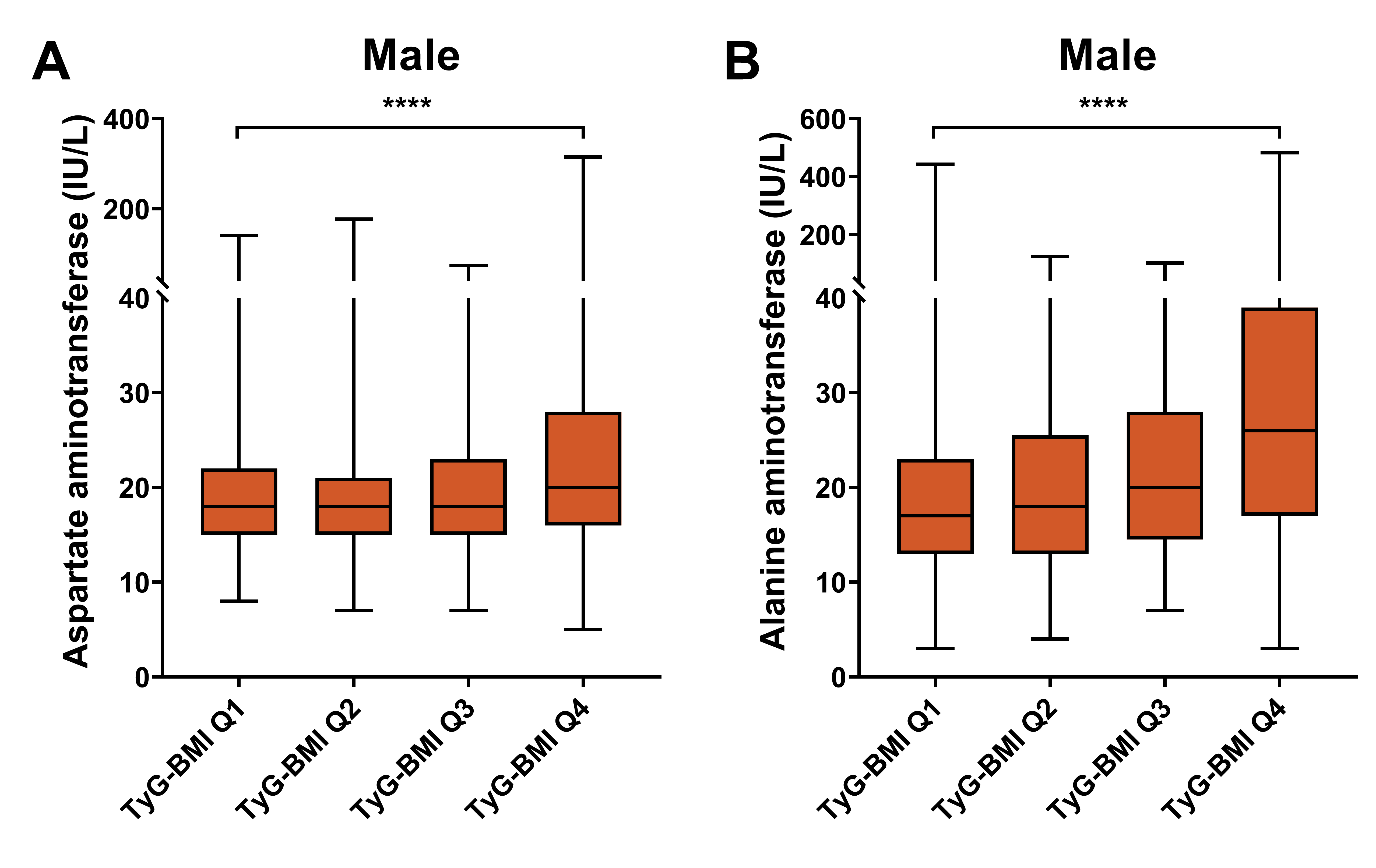


**Figure S7. Hepatic biomarkers of TyG-BMI subgroup in male T2D patients.**

Box plots showed the distribution of aspartate aminotransferase (AST) and alanine aminotransferase (ALT) levels across quartiles of TyG-BMI. (A) AST. The box plot showed the median, interquartile range, and outliers of AST measurements for each TyG-BMI quartile (Q1 to Q4). A significant increase in AST is observed with higher TyG-BMI quartiles. *****P* < 0.0001. (B) ALT. The box plot showed the median, interquartile range, and outliers of ALT measurements for each TyG-BMI quartile (Q1 to Q4). A significant increase in ALT is observed with higher TyG-BMI quartiles. *****P* < 0.0001.


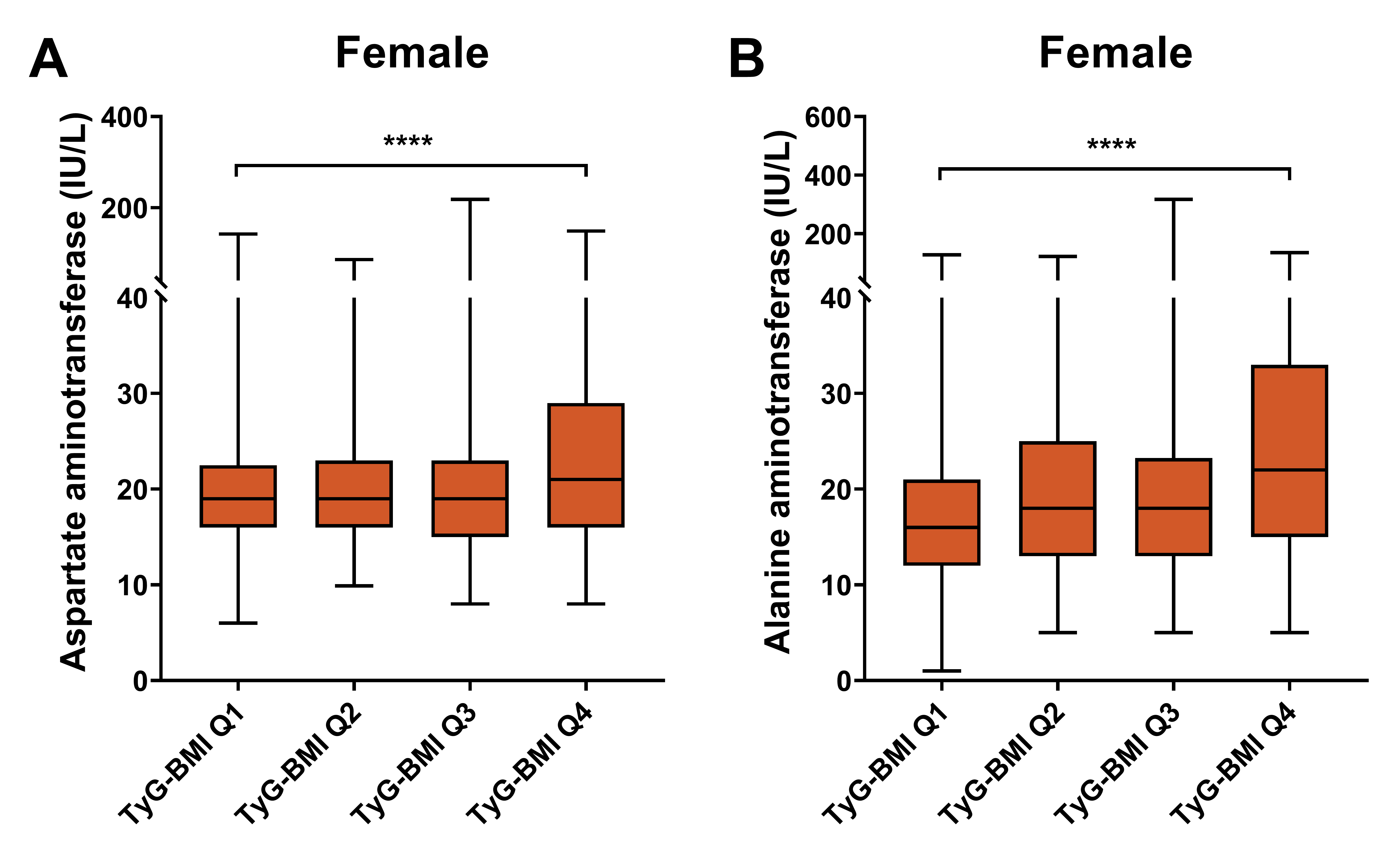


**Figure S8. Hepatic biomarkers of TyG-BMI subgroup in female T2D patients.**

Box plots showed the distribution of aspartate aminotransferase (AST) and alanine aminotransferase (ALT) levels across quartiles of TyG-BMI. (A) AST. The box plot showed the median, interquartile range, and outliers of AST measurements for each TyG-BMI quartile (Q1 to Q4). A significant increase in AST is observed with higher TyG-BMI quartiles. *****P* < 0.0001. (B) ALT. The box plot showed the median, interquartile range, and outliers of ALT measurements for each TyG-BMI quartile (Q1 to Q4). A significant increase in ALT is observed with higher TyG-BMI quartiles. *****P* < 0.0001.


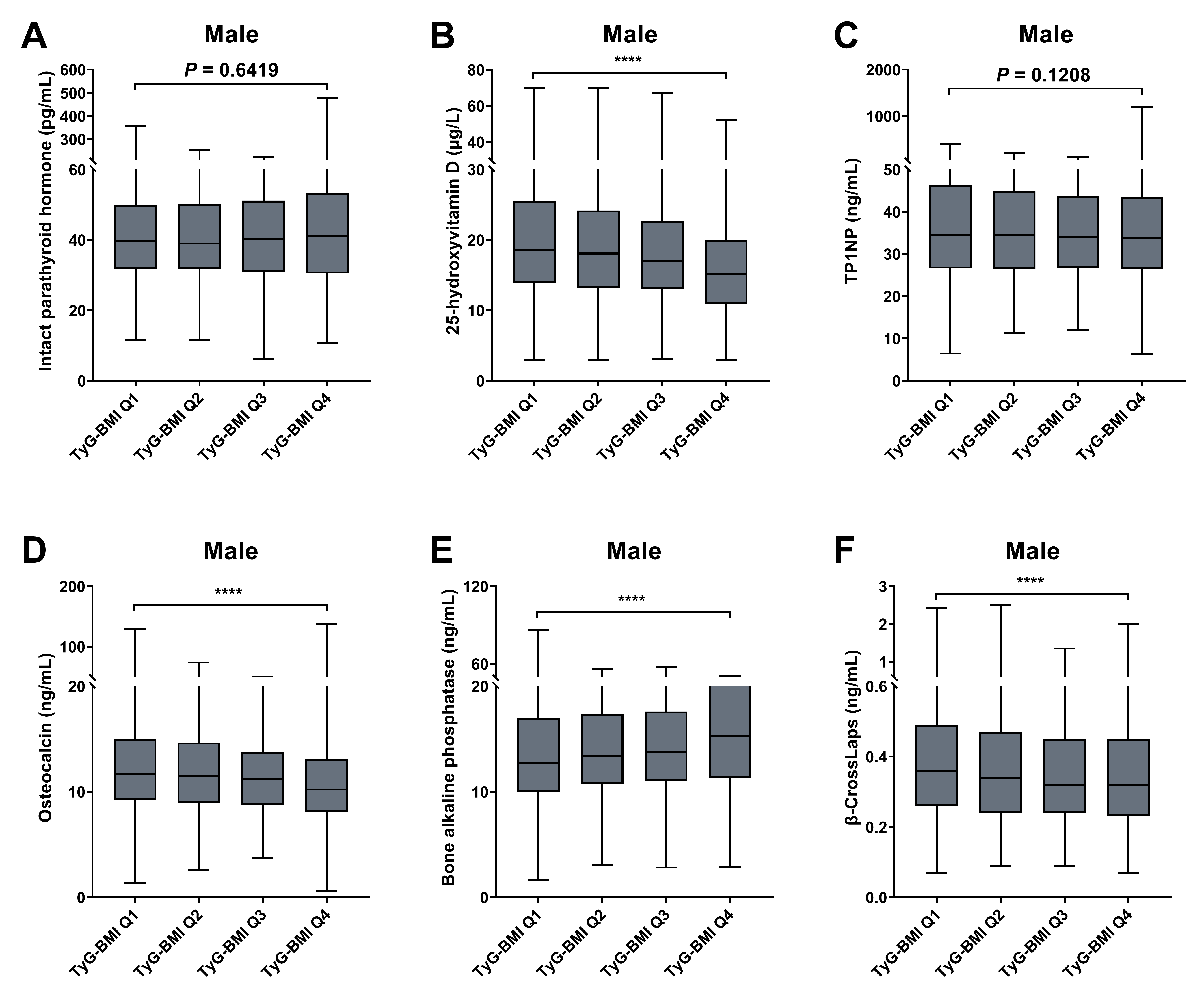


**Figure S9. Bone biomarkers of TyG-BMI subgroup in male T2D patients.**

Box plots showed the distribution of intact parathyroid hormone (IPTH), 25-hydroxyvitamin (25[OH]D), total procollagen type 1 N-terminal propeptide (TP1NP), osteocalcin; bone alkaline phosphatase (BALP) and β-CrossLaps levels across quartiles of TyG-BMI. (A) IPTH. The box plot showed the median, interquartile range, and outliers of IPTH measurements for each TyG-BMI quartile (Q1 to Q4). An increase in IPTH is observed with higher TyG-BMI quartiles. *P* = 0.3291. (B) 25(OH)D. The box plot showed the median, interquartile range, and outliers of 25(OH)D measurements for each TyG-BMI quartile (Q1 to Q4). A significant decrease in 25(OH)D is observed with higher TyG-BMI quartiles. *****P* < 0.0001. (C) TP1NP. The box plot showed the median, interquartile range, and outliers of TP1NP measurements for each TyG-BMI quartile (Q1 to Q4). A significant decrease in TP1NP is observed with higher TyG-BMI quartiles. *****P* < 0.0001. (D) Osteocalcin. The box plot showed the median, interquartile range, and outliers of osteocalcin measurements for each TyG-BMI quartile (Q1 to Q4). A significant decrease in osteocalcin is observed with higher TyG-BMI quartiles. *****P* < 0.0001. (E) BALP. The box plot showed the median, interquartile range, and outliers of BALP measurements for each TyG-BMI quartile (Q1 to Q4). A significant increase in BALP is observed with higher TyG-BMI quartiles. *****P* < 0.0001. (F) β-CrossLaps. The box plot showed the median, interquartile range, and outliers of β-CrossLaps measurements for each TyG-BMI quartile (Q1 to Q4). A significant decrease in β-CrossLaps is observed with higher TyG-BMI quartiles. *****P* < 0.0001.


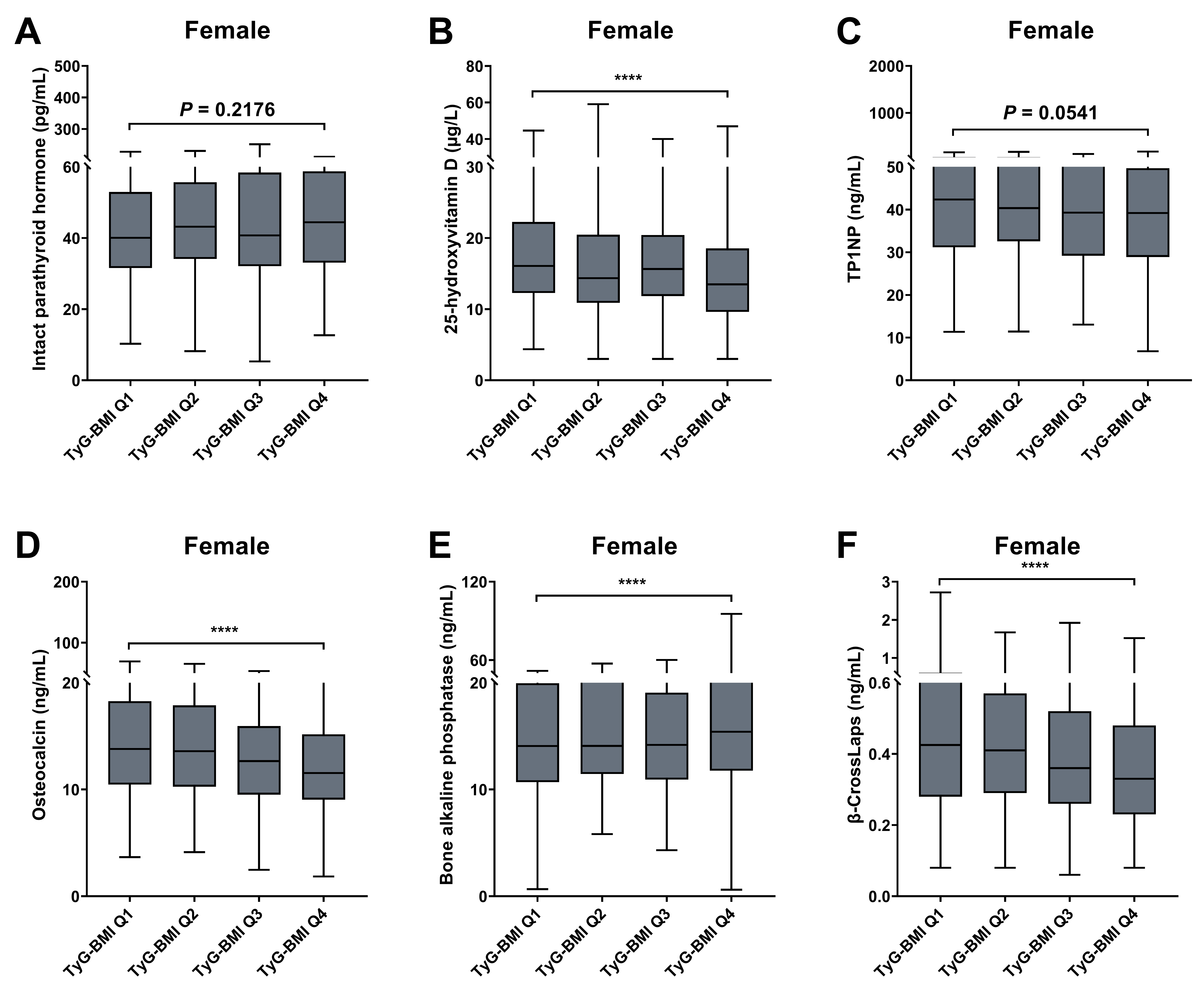


**Figure S10. Bone biomarkers of TyG-BMI subgroup in female T2D patients.**

Box plots showed the distribution of intact parathyroid hormone (IPTH), 25-hydroxyvitamin (25[OH]D), total procollagen type 1 N-terminal propeptide (TP1NP), osteocalcin; bone alkaline phosphatase (BALP) and β-CrossLaps levels across quartiles of TyG-BMI. (A) IPTH. The box plot showed the median, interquartile range, and outliers of IPTH measurements for each TyG-BMI quartile (Q1 to Q4). An increase in IPTH is observed with higher TyG-BMI quartiles. *P* = 0.3291. (B) 25(OH)D. The box plot showed the median, interquartile range, and outliers of 25(OH)D measurements for each TyG-BMI quartile (Q1 to Q4). A significant decrease in 25(OH)D is observed with higher TyG-BMI quartiles. *****P* < 0.0001. (C) TP1NP. The box plot showed the median, interquartile range, and outliers of TP1NP measurements for each TyG-BMI quartile (Q1 to Q4). A significant decrease in TP1NP is observed with higher TyG-BMI quartiles. *****P* < 0.0001. (D) Osteocalcin. The box plot showed the median, interquartile range, and outliers of osteocalcin measurements for each TyG-BMI quartile (Q1 to Q4). A significant decrease in osteocalcin is observed with higher TyG-BMI quartiles. *****P* < 0.0001. (E) BALP. The box plot showed the median, interquartile range, and outliers of BALP measurements for each TyG-BMI quartile (Q1 to Q4). A significant increase in BALP is observed with higher TyG-BMI quartiles. *****P* < 0.0001. (F) β-CrossLaps. The box plot showed the median, interquartile range, and outliers of β-CrossLaps measurements for each TyG-BMI quartile (Q1 to Q4). A significant decrease in β-CrossLaps is observed with higher TyG-BMI quartiles. *****P* < 0.0001.

**Table S1**. Biomarkers and their correlations with T2D complication risks in this study

| **Biomarker** | **Rationale for selection** | **Key pathophysiological link** |
| --- | --- | --- |
| ***Cardiovascular risk*** |  |  |
| Systolic/Diastolic pressure | Hypertension is a direct driver of CVD in T2D; strongly associated with insulin resistance | IR → RAAS activation → vasoconstriction → endothelial dysfunction |
| HDL | Inverse correlation with CVD risk; functional impairment (e.g., reduced cholesterol efflux) in T2D amplifies atherogenicity | TyG-BMI ↑ → HDL glycation → impaired reverse cholesterol transport |
| ***Renal risk*** |  |  |
| Uric acid | Hyperuricemia induces crystal nephropathy, tubulointerstitial inflammation, and intrarenal RAAS activation | TyG-BMI ↑ → purine metabolism dysregulation → uric acid overproduction |
| Sodium (Na) | Early marker of tubular dysfunction in diabetic nephropathy; hyponatremia reflects osmotic diuresis and solute wasting | Hyperglycemia → osmotic diuresis → Na^+^K^+^-ATPase impairment → urinary Na^+^ loss |
| ***Hepatic risk*** |  |  |
| AST/ALT | Gold-standard indicators of hepatocyte injury; elevated in NAFLD/NASH progression | TyG-BMI ↑ → hepatic lipid accumulation → mitochondrial stress → enzyme leakage |
| ***Bone risk*** |  |  |
| 25(OH)D | Deficiency exacerbates insulin resistance and impairs osteoblast-mediated mineralization | TyG-BMI ↑ → visceral adiposity ↑ → vitamin D sequestration in fat → functional deficiency |
| IPTH | Secondary hyperparathyroidism from vitamin D deficiency drives bone resorption | Low 25(OH)D → ↑ PTH → RANKL activation → osteoclastogenesis |
| TP1NP | Direct marker of osteoblast collagen synthesis; reduced in diabetic osteopathy | AGEs ↓ osteoblast lifespan → ↓ collagen type 1 production |
| Osteocalcin | Insulin-sensitizing osteokine; uncarboxylated form (ucOC) regulates β-cell function | Hyperglycemia ↓ osteocalcin carboxylation → disrupted bone-pancreas crosstalk |
| Bone ALP | Reflects osteoblast activity; paradoxically elevated in T2D due to compensatory proliferation | IR → oxidative stress → osteoblast apoptosis → compensatory ALP release |
| β-CrossLaps | Collagen degradation product; indicates osteoclast resorption activity | Adiponectin ↑ (from IR) ↓ RANKL → suppressed resorption → low β-CrossLaps |

**Table S3**. Characteristics of age and bone, cardiovascular, renal and hepatic biomarkers of male T2D patients in different TyG-BMI categories

| **Characteristic** | **Q1, N = 471** | **Q2, N = 471** | **Q3, N = 471** | **Q4, N = 470** | **p-value** |
| --- | --- | --- | --- | --- | --- |
| Age |  |  |  |  | <0.0001 |
| Mean ± SD | 60 ± 12 | 59 ± 10 | 56 ± 11 | 49 ± 12 |  |
| Median (IQR) | 62 (55, 68) | 60 (52, 67) | 57 (49, 64) | 50 (40, 58) |  |
| Range | 14, 86 | 24, 89 | 17, 85 | 15, 85 |  |
| **Bone biomarker** |  |  |  |  |  |
| Intact parathyroid hormone (pg/mL) |  |  |  |  | 0.6419 |
| Mean ± SD | 44 ± 24 | 44 ± 22 | 45 ± 23 | 46 ± 32 |  |
| Median (IQR) | 40 (32, 50) | 39 (32, 50) | 40 (31, 51) | 41 (31, 53) |  |
| Range | 11, 358 | 11, 253 | 6, 223 | 11, 476 |  |
| 25-hydroxyvitamin D (µg/L) |  |  |  |  | <0.0001 |
| Mean ± SD | 20 ± 9 | 19 ± 8 | 18 ± 7 | 16 ± 7 |  |
| Median (IQR) | 19 (14, 25) | 18 (13, 24) | 17 (13, 23) | 15 (11, 20) |  |
| Range | 3, 70 | 3, 70 | 3, 67 | 3, 52 |  |
| TP1NP (ng/mL) |  |  |  |  | 0.1208 |
| Mean ± SD | 41 ± 35 | 38 ± 18 | 36 ± 14 | 39 ± 57 |  |
| Median (IQR) | 35 (27, 46) | 35 (26, 45) | 34 (27, 44) | 34 (27, 43) |  |
| Range | 6, 400 | 11, 199 | 12, 118 | 6, 1,200 |  |
| Osteocalcin (ng/mL) |  |  |  |  | <0.0001 |
| Mean ± SD | 13.2 ± 8.8 | 12.4 ± 5.5 | 11.8 ± 4.8 | 11.5 ± 7.9 |  |
| Median (IQR) | 11.7 (9.3, 15.0) | 11.5 (8.9, 14.6) | 11.2 (8.8, 13.8) | 10.2 (8.1, 13.0) |  |
| Range | 1.4, 129.4 | 2.6, 74 | 3.7, 50.5 | 0.6, 138.2 |  |
| Bone alkaline phosphatase (ng/mL) |  |  |  |  | <0.0001 |
| Mean ± SD | 14 ± 7 | 14 ± 6 | 15 ± 6 | 16 ± 7 |  |
| Median (IQR) | 13 (10, 17) | 13 (11, 17) | 14 (11, 18) | 15 (11, 20) |  |
| Range | 2, 86 | 3, 56 | 3, 57 | 3, 51 |  |
| β-CrossLaps (ng/mL) |  |  |  |  | <0.0001 |
| Mean ± SD | 0.41 ± 0.26 | 0.38 ± 0.21 | 0.36 ± 0.18 | 0.37 ± 0.23 |  |
| Median (IQR) | 0.36 (0.26, 0.49) | 0.34 (0.24, 0.46) | 0.32 (0.24, 0.45) | 0.32 (0.23, 0.45) |  |
| Range | 0.07, 2.43 | 0.09, 2.50 | 0.09, 1.35 | 0.07, 2.00 |  |
| **Cardiovascular biomarker** |  |  |  |  |  |
| Systolic pressure (mmHg) |  |  |  |  | <0.0001 |
| Mean ± SD | 126 ± 18 | 132 ± 19 | 133 ± 17 | 134 ± 18 |  |
| Median (IQR) | 125 (114, 140) | 131 (119, 145) | 131 (120, 145) | 132 (123, 145) |  |
| Range | 80, 185 | 89, 202 | 91, 208 | 58, 203 |  |
| Diastolic pressure (mmHg) |  |  |  |  | <0.0001 |
| Mean ± SD | 79 ± 10 | 84 ± 10 | 86 ± 10 | 90 ± 11 |  |
| Median (IQR) | 80 (73, 86) | 85 (78, 92) | 87 (79, 94) | 90 (83, 98) |  |
| Range | 43, 111 | 48, 116 | 56, 121 | 61, 146 |  |
| High density lipoprotein (mmol/L) |  |  |  |  | <0.0001 |
| Mean ± SD | 1.30 ± 0.31 | 1.17 ± 0.23 | 1.10 ± 0.24 | 0.98 ± 0.21 |  |
| Median (IQR) | 1.26 (1.07, 1.48) | 1.16 (1.01, 1.30) | 1.08 (0.94, 1.24) | 0.95 (0.84, 1.12) |  |
| Range | 0.55, 2.45 | 0.64, 2.55 | 0.57, 2.40 | 0.43, 1.67 |  |
| **Renal biomarker** |  |  |  |  |  |
| Uric acid (µmol/L) |  |  |  |  | <0.0001 |
| Mean ± SD | 305 ± 76 | 322 ± 75 | 340 ± 84 | 372 ± 97 |  |
| Median (IQR) | 299 (253, 351) | 317 (275, 371) | 336 (282, 388) | 369 (304, 432) |  |
| Range | 74, 630 | 99, 593 | 142, 688 | 36, 825 |  |
| Na (mmol/L) |  |  |  |  | <0.0001 |
| Mean ± SD | 139.45 ± 2.58 | 139.04 ± 2.47 | 138.67 ± 2.40 | 137.96 ± 2.80 |  |
| Median (IQR) | 139.50 (138.00, 141.05) | 139.10 (137.60, 140.70) | 138.70 (137.30, 140.30) | 138.00 (136.10, 140.00) |  |
| Range | 126.00, 148.50 | 130.10, 147.10 | 129.60, 146.00 | 122.90, 145.90 |  |
| **Hepatic biomarker** |  |  |  |  |  |
| Aspartate aminotransferase (IU/L) |  |  |  |  | <0.0001 |
| Mean ± SD | 19 ± 9 | 19 ± 11 | 20 ± 8 | 25 ± 21 |  |
| Median (IQR) | 18 (15, 22) | 18 (15, 21) | 18 (15, 23) | 20 (16, 28) |  |
| Range | 8, 141 | 7, 177 | 7, 75 | 5, 315 |  |
| Alanine aminotransferase (IU/L) |  |  |  |  | <0.0001 |
| Mean ± SD | 20 ± 22 | 21 ± 14 | 23 ± 13 | 35 ± 36 |  |
| Median (IQR) | 17 (13, 23) | 18 (13, 25) | 20 (15, 28) | 26 (17, 39) |  |
| Range | 3, 443 | 4, 125 | 7, 102 | 3, 482 |  |

*Note*: The full names of abbreviations are: TP1NP – Total procollagen type 1 N-terminal propeptide; Na - sodium.

**Table S4**. Characteristics of age and bone, cardiovascular, renal and hepatic biomarkers of female T2D patients in different TyG-BMI categories

| **Characteristic** | **Q1, N = 274** | **Q2, N = 274** | **Q3, N = 275** | **Q4, N = 275** | **p-value** |
| --- | --- | --- | --- | --- | --- |
| Age |  |  |  |  | <0.0001 |
| Mean ± SD | 61 ± 11 | 61 ± 11 | 62 ± 12 | 58 ± 13 |  |
| Median (IQR) | 62 (56, 68) | 63 (56, 69) | 63 (56, 69) | 61 (52, 68) |  |
| Range | 20, 87 | 14, 88 | 15, 90 | 22, 83 |  |
| **Bone biomarker** |  |  |  |  |  |
| Intact parathyroid hormone (pg/mL) |  |  |  |  | 0.2176 |
| Mean ± SD | 45 ± 22 | 48 ± 27 | 47 ± 23 | 49 ± 25 |  |
| Median (IQR) | 40 (32, 53) | 43 (34, 56) | 41 (32, 58) | 44 (33, 59) |  |
| Range | 10, 228 | 8, 231 | 5, 252 | 13, 212 |  |
| 25-hydroxyvitamin D (µg/L) |  |  |  |  | <0.0001 |
| Mean ± SD | 17 ± 8 | 16 ± 8 | 17 ± 7 | 15 ± 7 |  |
| Median (IQR) | 16 (12, 22) | 14 (11, 20) | 16 (12, 20) | 13 (10, 18) |  |
| Range | 4, 45 | 3, 59 | 3, 40 | 3, 47 |  |
| TP1NP (ng/mL) |  |  |  |  | 0.0541 |
| Mean ± SD | 46 ± 22 | 45 ± 21 | 42 ± 18 | 42 ± 21 |  |
| Median (IQR) | 42 (31, 55) | 41 (33, 54) | 39 (29, 50) | 39 (29, 50) |  |
| Range | 11, 157 | 11, 167 | 13, 121 | 7, 173 |  |
| Osteocalcin (ng/mL) |  |  |  |  | <0.0001 |
| Mean ± SD | 15.0 ± 7.8 | 14.9 ± 7.5 | 13.6 ± 6.2 | 12.5 ± 5.6 |  |
| Median (IQR) | 13.8 (10.5, 18.3) | 13.6 (10.3, 17.8) | 12.6 (9.6, 15.9) | 11.6 (9.1, 15.1) |  |
| Range | 3.7, 69.2 | 4.1, 65.4 | 2.5, 53.3 | 1.8, 43.4 |  |
| Bone alkaline phosphatase (ng/mL) |  |  |  |  | <0.0001 |
| Mean ± SD | 16 ± 8 | 17 ± 8 | 16 ± 8 | 18 ± 10 |  |
| Median (IQR) | 14 (11, 20) | 14 (12, 20) | 14 (11, 19) | 15 (12, 21) |  |
| Range | 1, 52 | 6, 57 | 4, 60 | 1, 95 |  |
| β-CrossLaps (ng/mL) |  |  |  |  | <0.0001 |
| Mean ± SD | 0.48 ± 0.30 | 0.46 ± 0.25 | 0.42 ± 0.23 | 0.38 ± 0.20 |  |
| Median (IQR) | 0.43 (0.28, 0.61) | 0.41 (0.29, 0.57) | 0.36 (0.26, 0.52) | 0.33 (0.23, 0.48) |  |
| Range | 0.08, 2.72 | 0.08, 1.67 | 0.06, 1.92 | 0.08, 1.52 |  |
| **Cardiovascular biomarker** |  |  |  |  |  |
| Systolic pressure (mmHg) |  |  |  |  | <0.0001 |
| Mean ± SD | 131 ± 19 | 136 ± 20 | 137 ± 19 | 138 ± 20 |  |
| Median (IQR) | 128 (117, 144) | 135 (121, 149) | 136 (124, 149) | 138 (123, 150) |  |
| Range | 82, 196 | 91, 192 | 95, 192 | 93, 201 |  |
| Diastolic pressure (mmHg) |  |  |  |  | <0.0001 |
| Mean ± SD | 79 ± 10 | 82 ± 11 | 82 ± 10 | 85 ± 11 |  |
| Median (IQR) | 78 (72, 85) | 82 (74, 90) | 82 (75, 89) | 84 (77, 93) |  |
| Range | 53, 114 | 49, 113 | 57, 113 | 59, 135 |  |
| High density lipoprotein (mmol/L) |  |  |  |  | <0.0001 |
| Mean ± SD | 1.49 ± 0.35 | 1.35 ± 0.31 | 1.26 ± 0.26 | 1.19 ± 0.26 |  |
| Median (IQR) | 1.46 (1.24, 1.70) | 1.32 (1.15, 1.53) | 1.24 (1.08, 1.41) | 1.15 (1.00, 1.34) |  |
| Range | 0.85, 2.83 | 0.44, 2.46 | 0.73, 2.24 | 0.48, 1.96 |  |
| **Renal biomarker** |  |  |  |  |  |
| Uric acid (µmol/L) |  |  |  |  | <0.0001 |
| Mean ± SD | 264 ± 76 | 287 ± 81 | 305 ± 87 | 322 ± 87 |  |
| Median (IQR) | 257 (217, 311) | 282 (234, 331) | 294 (248, 348) | 319 (259, 373) |  |
| Range | 7, 541 | 105, 593 | 5, 642 | 141, 691 |  |
| Na (mmol/L) |  |  |  |  | <0.0001 |
| Mean ± SD | 139.70 ± 2.75 | 139.43 ± 2.62 | 139.18 ± 2.63 | 138.59 ± 2.83 |  |
| Median (IQR) | 140.10 (138.10, 141.40) | 139.40 (137.70, 141.10) | 139.40 (137.83, 140.90) | 138.60 (136.70, 140.40) |  |
| Range | 130.30, 146.70 | 129.60, 146.70 | 124.30, 146.50 | 128.00, 151.90 |  |
| **Hepatic biomarker** |  |  |  |  |  |
| Aspartate aminotransferase (IU/L) |  |  |  |  | <0.0001 |
| Mean ± SD | 21 ± 13 | 21 ± 8 | 21 ± 15 | 25 ± 16 |  |
| Median (IQR) | 19 (16, 22) | 19 (16, 23) | 19 (15, 23) | 21 (16, 29) |  |
| Range | 6, 142 | 10, 86 | 8, 218 | 8, 149 |  |
| Alanine aminotransferase (IU/L) |  |  |  |  | <0.0001 |
| Mean ± SD | 19 ± 14 | 20 ± 12 | 22 ± 22 | 28 ± 20 |  |
| Median (IQR) | 16 (12, 21) | 18 (13, 25) | 18 (13, 23) | 22 (15, 33) |  |
| Range | 1, 128 | 5, 122 | 5, 317 | 5, 135 |  |

*Note*: The full names of abbreviations are: TP1NP – Total procollagen type 1 N-terminal propeptide; Na - sodium.
